# A recurrence-based approach for validating structural variation using long-read sequencing technology

**DOI:** 10.1093/gigascience/gix061

**Published:** 2017-07-19

**Authors:** Xuefang Zhao, Alexandra M. Weber, Ryan E. Mills

**Affiliations:** 1 Department of Computational Medicine and Bioinformatics, University of Michigan, 100 Washtenaw Ave, Ann Arbor, MI 48109, USA; 2 Department of Human Genetics, University of Michigan, 1241 Catherine St, Ann Arbor, MI 48109, USA

**Keywords:** structural variation, copy number variation, sequence analysis

## Abstract

Although numerous algorithms have been developed to identify structural variations (SVs) in genomic sequences, there is a dearth of approaches that can be used to evaluate their results. This is significant as the accurate identification of structural variation is still an outstanding but important problem in genomics. The emergence of new sequencing technologies that generate longer sequence reads can, in theory, provide direct evidence for all types of SVs regardless of the length of the region through which it spans. However, current efforts to use these data in this manner require the use of large computational resources to assemble these sequences as well as visual inspection of each region. Here we present VaPoR, a highly efficient algorithm that autonomously validates large SV sets using long-read sequencing data. We assessed the performance of VaPoR on SVs in both simulated and real genomes and report a high-fidelity rate for overall accuracy across different levels of sequence depths. We show that VaPoR can interrogate a much larger range of SVs while still matching existing methods in terms of false positive validations and providing additional features considering breakpoint precision and predicted genotype. We further show that VaPoR can run quickly and efficiency without requiring a large processing or assembly pipeline. VaPoR provides a long read–based validation approach for genomic SVs that requires relatively low read depth and computing resources and thus will provide utility with targeted or low-pass sequencing coverage for accurate SV assessment. The VaPoR Software is available at: https://github.com/mills-lab/vapor.

## Background

Structural variants (SVs) are 1 of the major forms of genetic variation in humans and have been revealed to play important roles in numerous diseases including cancers and neurological disorders [1, 2]. Various approaches have been developed and applied to paired-end sequencing to detect SVs in whole genomes [3–6]; however, individual algorithms often exhibit complementary strengths that sometimes lead to disagreements as to the precise structure of the underlying variant. The emergence of long-read sequencing technology, such as Single Molecule Real-Time (SMRT) sequencing from Pacific Biosciences (PacBio) [7, 8], can deliver reads ranging from several to hundreds of kilobases and provide direct evidence for the presence of an SV. Current strategies make use of *de novo* assembly to create long contigs with minimized error rate [9–11] and then predict SVs, typically with single base resolution, through direct comparison of the assembly against the reference. Though such approaches are powerful, they require both a very high sequencing depth and significant computing power and are currently impracticable for many ongoing research studies.

The additional information obtained from using long reads can still be leveraged to improve variant calling, however. Indeed, such approaches have already been implemented to combine high-depth Illumina sequencing with lower-depth PacBio reads to improve error correction and variant calling in the context of *de novo* genome assembly [12]. With structural variation, the current state of the art is to use long reads to manually assess potential SVs using subsequent recurrence (dot) plots [13], where the sequences are compared against the reference through a fixed size sliding window (k-mer) and the matches are plotted for visual inspection. The k-mer method is of higher robustness compared to direct sequences comparison [14], which is why these types of dot plots have been used for decades to examine the specific features of sequence alignments [15]. However, they require manual curation and, coupled with the computational costs of sequence assembly, are time-consuming and inefficient at scale for the high-throughput validation of large sets of SVs.

Here, we present a high-speed long read–based assessment tool, VaPoR, that investigates and scores each provided SV prediction by autonomously analyzing the recurrence of k-mers within a local read against both an unmodified reference sequence at that loci as well as a rearranged reference pertaining to the predicted SV structure. A positive score of each read on the altered reference, normalized against the score of the read on the original reference, supports the predicted structure. A baseline model is constructed as well by interrogating the reference sequence against itself at the query location. We show that our approach can quickly and accurately distinguish true from false positive predictions of both simple and complex SVs as well as their underlying genotypes and that it is also able to assess the breakpoint accuracy of individual algorithms.

## Data Description

### Simulated data

Non-overlapping simple deletions, inversions, insertions, and duplications, as well as complex structural variants, as previously categorized [5], were independently incorporated into GRCh38 in both heterozygous and homozygous states, excluding regions of the genome that are known to be difficult to assess, as described by the ENCODE project [16]. Detailed descriptions of each simulated SV type simulated are summarized in Supplementary Tables S1 and S2. We applied PBSIM (PBSIM, RRID:SCR_002512) [17] to simulate the modified reference sequences to different read depths, ranging from ×2 to ×70, with a parameters difference-ratio of 5:75:20, length-mean of 12 000, accuracy-mean of 0.85, and *model_qc model_qc_clr*. Simulated data can be obtained from the author's institution [18] and via the *GigaScience* repository, *GigaDB* [19].

### Real data

We applied VaPoR to a set of diverse samples (HG00513 from CHS, HG00731 and HG00732 from PUR, NA19238 and NA19239 from YRI) that were initially sequenced by the 1000 Genomes Project and for which a high-quality set of SVs were reported in the final phase of the project [20]. These samples were recently re-sequenced using PacBio to ×20 coverage, and they therefore provide a platform for assessing VaPoR on known data. The 1000 Genomes Project (1KGP) Phase 3 data were obtained from 1KGP’s Integrated SV Map [21] and lifted over to GRCh38. PacBio sequence data were obtained from 1KGP’s HGSV SV Discovery [22].

We have also compared VaPoR against the long-read validation approach developed by Layer et al. [3], which requires both PacBio and Moleculo long sequences for full evaluation of SVs. These comparisons made use of NA12878, 1 of few samples that have been sequenced with various technologies including Illumina NGS, PacBio, and Moleculo with a truth SV set included in the 1KGP Phase 3 report. The software for the long-read validation approach was obtained from github's Long-Read Validation page [23]. The PacBio and the Moleculo sequences of this individual were obtained from 1KGP’s SI [24] and Alignment pages [25], respectively.

## Results

We assessed the performance of VaPoR on both simulated sequences and real genomes from the 1000 Genomes Project to assess the following characteristics: sensitivity and false discovery rate on validating structural variants in simple and complex structures; sensitivity of VaPoR on validating different levels of predicted breakpoint efficacy; stratification of VaPoR scores by genotype; and time and computational cost of VaPoR.

### VaPoR on simulated data

We applied VaPoR to simulated simple deletions, inversions, insertions, and duplications as well as complex structural variants and first assessed the proportion of SVs that VaPoR is capable of interrogating (i.e., passed VaPoR QC). We found that VaPoR can successfully evaluate >80% of insertions, >85% of deletion-duplications, and >90% of SVs in all other categories when the read depth is ×10 or higher. We then assessed the sensitivity and false discovery rate (FDR) at different VaPoR score cutoffs and found that a sensitivity >90% is achieved for most SV types across a wide range of read depths while maintaining a false discovery rate <10% at a VaPoR score cutoff of 0.15 (Fig. 2; Supplementary Figs S1 and S2). We further observed that there were no significant changes of sensitivity or false discovery rate once the read depth was at or above ×20 and consistent across different SV types (Fig. 3; Supplementary Table S3).

### VaPoR on 1000 Genomes Project samples

We next examined SVs reported on chr1 of 5 diverse individuals from the 1000 Genomes Project [26] to assess the sensitivity of VaPoR on real genomes (Table 1), with 197–258 SVs reported per individual in Phase 3 of the project. We first observed that >95% of deletions and insertions could be successfully evaluated by VaPoR. For inversions, there were a limited number of events reported but at maximum only 1 event failed the VaPoR quality control per individual. Moreover, we observed 3–8% of deletions and insertions that are 10 Kb or larger in size across the individuals. Such events were rarely fully covered by long sequences according their length distribution (Supplementary Fig. S3) and were assessed through the “large variants assessment” module implemented in VaPoR (Methods, Supplementary Fig. S4), out of which 100% were successfully evaluated. A sensitivity of >90% was achieved for deletions (Fig. 4a) and >80% for insertions (Fig. 4b) at the recommended cutoff of 0.15.

**Table 1:** Sensitivity and false discovery rate of different SV types

	Deletion	Insertion	Inversion
Sample	Sens/FDR	Sens/FDR	Sens/FDR
HG00513	0.96/0.00 (0.94^a^)	0.80/0.05 (0.93)	0.50/0.00 (0.71)
HG00731	0.94/0.00 (0.96)	0.85/0.07 (0.97)	0.60/0.00 (1.00)
HG00732	0.92/0.00 (0.98)	0.92/0.08 (0.96)	0.33/0.00 (0.86)
NA19238	0.90/0.00 (0.93)	0.88/0.10 (0.96)	1.00/0.00 (1.00)
NA19239	0.87/0.02 (0.95)	0.73/0.09 (0.96)	0.33/0.00 (1.00)

^a^The proportion of SVs that passed VaPoR QC, as listed in brackets, are counted for events on chr1 and chr2 together.

To examine the false validation rate of VaPoR, we modified reported events on chr2 to appear at the same coordinates on chr1 and assessed them as though they were real events using the same sequence data set. VaPoR validated very few deletions or inversions and <10% of insertions. We further compared VaPoR against a long-read validation approach developed in conjunction with Lumpy (Lumpy, RRID:SCR_003253) [3] using SVs on chr1 of NA12878 reported by the 1kGP Phase 3. VaPoR achieved a sensitivity of 72% for deletions and 86% for insertions, while the Lumpy-associated approach was only able to assess 11% and 0%, respectively. Both approaches exhibited a very low false validation rate when synthetically assigning the variants to chr2, with 0 for all SV types by the Layer et al. approach and varying between 0% and 2.5% for VaPoR (Supplementary Table S4).

### SV breakpoint validation and accuracy

One of the outstanding challenges of SV discovery is the precise determination of its location at nucleotide resolution. Many short-read algorithms can correctly identify the presence of an SV but report uncertainty at the breakpoints, as can be observed by the reported median confidence intervals of +/−85 bp across all events in the 1KGP Phase 3 set [20]. We therefore assessed the performance of VaPoR to validate SVs with varying degrees of breakpoint accuracy by artificially shifting the coordinates of simulated SVs (Supplementary Figs S5 and S6) and the Phase 3 SVs from the 1000 Genomes samples (Fig. 4c and d) by –1000 to 1000 base pairs and re-assessing the new positions with VaPoR. Using default parameters, VaPoR exhibited a robust validation score, up to approximately 200 bp overall, with some slight differences observed between different SV types. We note that this delineation is partially dependent on the length of the flanking sequence selected as larger flanking sequences would allow for larger breakpoint offsets depending on user preference. SVs with confidence intervals bounding expected breakpoint locations can be also be systematically assessed using subsequent VaPoR application with offset breakpoints to identify the positions that exhibit the highest score.

### Discrimination of SV types and genotypes

We identified a small number of SVs in the high-quality 1000 Genomes set that did not validate with VaPoR. Previous studies have shown that complex rearrangements are often misclassified as simple structural changes [5, 13], and indeed, upon manual inspection, these appeared to consist of multiple connected rearrangements. For example, we observed a reported inversion in HG00513 and NA19239 on chromosome 1 (chr1:239952707–239953529) that was invalidated by VaPoR; an investigation into the long reads aligned in the region showed the signature of an inverted duplication (Fig. 5a) that, when incorporated into a modified reference location, matched almost exactly with the read sequence (Fig. 5b).

We further explored the distribution of VaPoR scores for this region and others across the sample set and observed clear delineations between allelic copy number when fitted with a Gaussian mixture model, allowing for the generation of genotype likelihoods for each site (Fig. 5c). These tracked with our expected genotypes for the inverted duplication on chr1 across the 5 individuals queried while showing no support for the originally predicted inversion (Fig. 5d). This shows that VaPoR is not only able to accurately genotype variants but can also distinguish between similar but distinct SV predictions in the same region.

Using these data, we implemented a genotyping module as an option for users to assess predicted genotypes with those derived using long reads. We compared the genotype of deletions and inversions reported by the 1000 Genomes Phase 3 to the VaPoR genotypes at those loci and observed a non-reference genotype concordance of 0.83 (Supplementary Table S5). The manual visual inspection of regions with discordant genotypes using both the Illumina WGS and PacBio sequence alignments in IGV [27] showed the VaPoR genotypes to be consistently correct in such cases. An updated non-reference genotype concordance of 0.95 was achieved after we integrated these manual inspections into the 1000 Genomes set.

### Runtime and efficiency

The computation runtime of VaPoR was assessed using 2 Intel Xeon Intel Xeon E7–4860 processors with 4 GB of RAM each on both simulated and real genomes. The runtime of the simulated event was observed to increase linearly with read depth (Supplementary Fig. S7). For events sequenced up to ×20, VaPoR takes ∼3 seconds to assess a simple SV and ∼5 seconds for a complex event. The assessment of real samples sequenced at ×20 required ∼1.4 seconds to assess a simple deletion or insertion and ∼6 seconds for an inversion (Supplementary Table S6), with a full genome analysis consisting of ∼3000 SVs larger than 50 bp, taking 2 CPU hours on average.

## Discussion

Here we present an automated assessment approach, named VaPoR, for exploring various features of predicted genomic structural variants using long-read sequencing data. VaPoR directly compares the input reads with the reference sequences with relatively straightforward computational metrics, thus achieving high efficiency in both run time and computing cost. VaPoR exhibits high sensitivity and specificity in both simulated and real genomes, with the capability of discriminating partially resolved SVs either consisting of similar but incorrect SV types at the same location or correct SVs with offset breakpoints. Furthermore, we show that VaPoR performs well at low read depths (×5–10), thus providing the option of systematically assessing large-scale SVs at a lower sequencing cost.

## Methods

### VaPoR workflow

**Figure 1:**
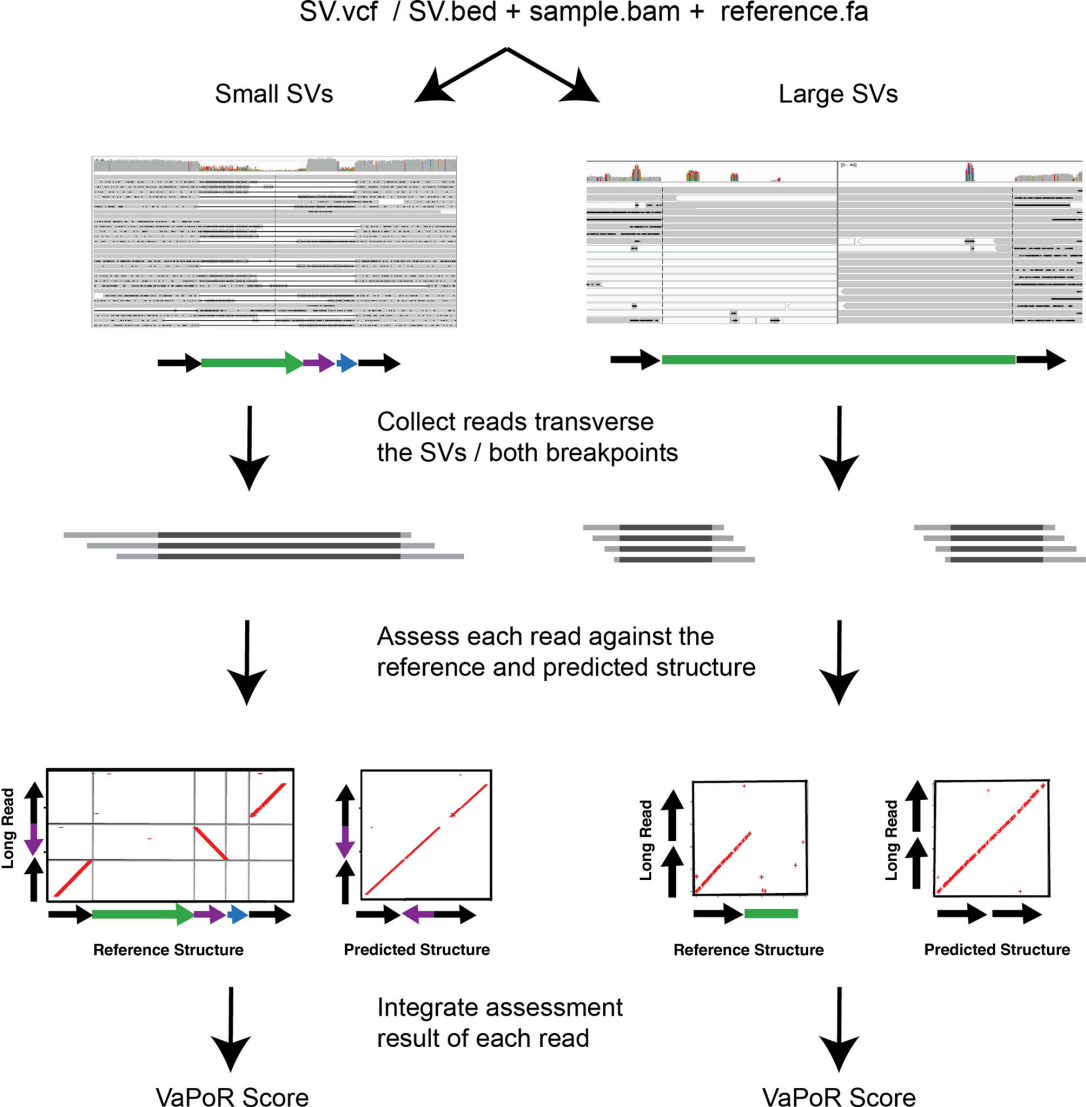
Flowchart describing the VaPoR algorithm. As input, the algorithm requires a set of structural variants in either VCF or BED format, a series of long reads and/or sequence contigs in BAM format, and the corresponding reference sequence. VaPoR then interrogates each variant individually at its corresponding reference location, assesses the quality of the region, and assigns a score.

VaPoR takes in aligned sequence reads in BAM format and predicted SVs (>50 bp) in various formats including VCF and BED. SVs are evaluated by comparing long reads that traverse the reported position of the event against reference sequences in 2 formats: (i) the original human reference to which the sample is aligned and (ii) a modified reference sequence altered to match the predicted structural rearrangement. A recurrence matrix is then derived by sliding a fixed-size window (k-mer) with a 1-bp step through each read to mark positions where the read sequence and reference are identical. The matching patterns are then assessed as to the validity of the SV, and a validation score is reported. Given the large variance of SV lengths, each SV is stratified into 1 of 2 groups: smaller SVs that can be completely encompassed within multiple (>10 by default) long sequences and larger events that are too big to fall within individual reads but for which the breakpoint regions can be assessed. Each class of SV is interrogated with different statistical models, as described below. The VaPoR workflow is briefly summarized in Fig. 1.

**Figure 2:**
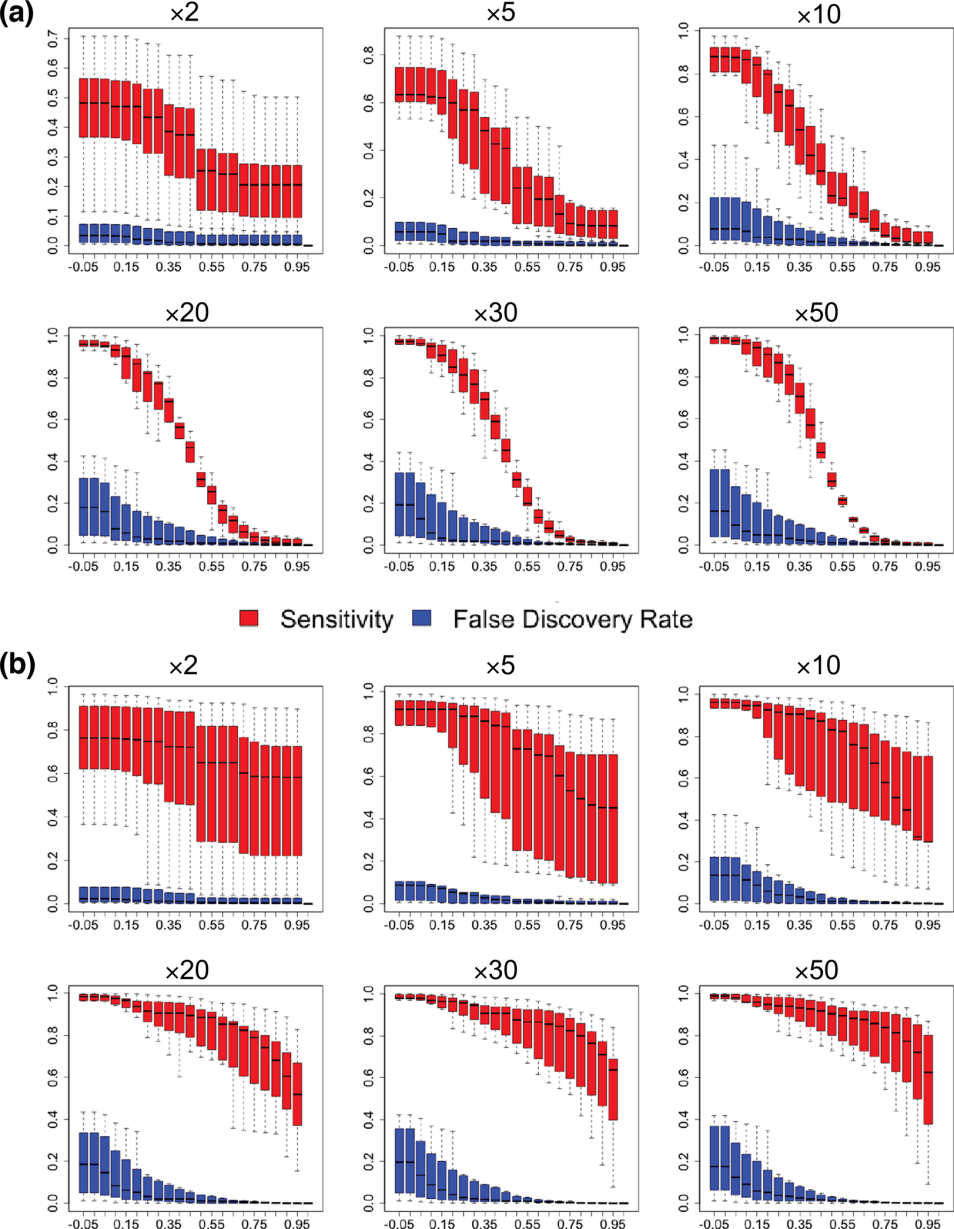
Accuracy of VaPoR on simulated heterozygous and homozygous SVs at varying degrees of sequence coverage and VaPoR score cutoffs. The validation success rate is shown for simulated true positive (red) and false positive (blue) variants in both (**a**) heterozygous and (**b**) homozygous states from ×2 to ×50 genome coverage.

**Figure 3:**
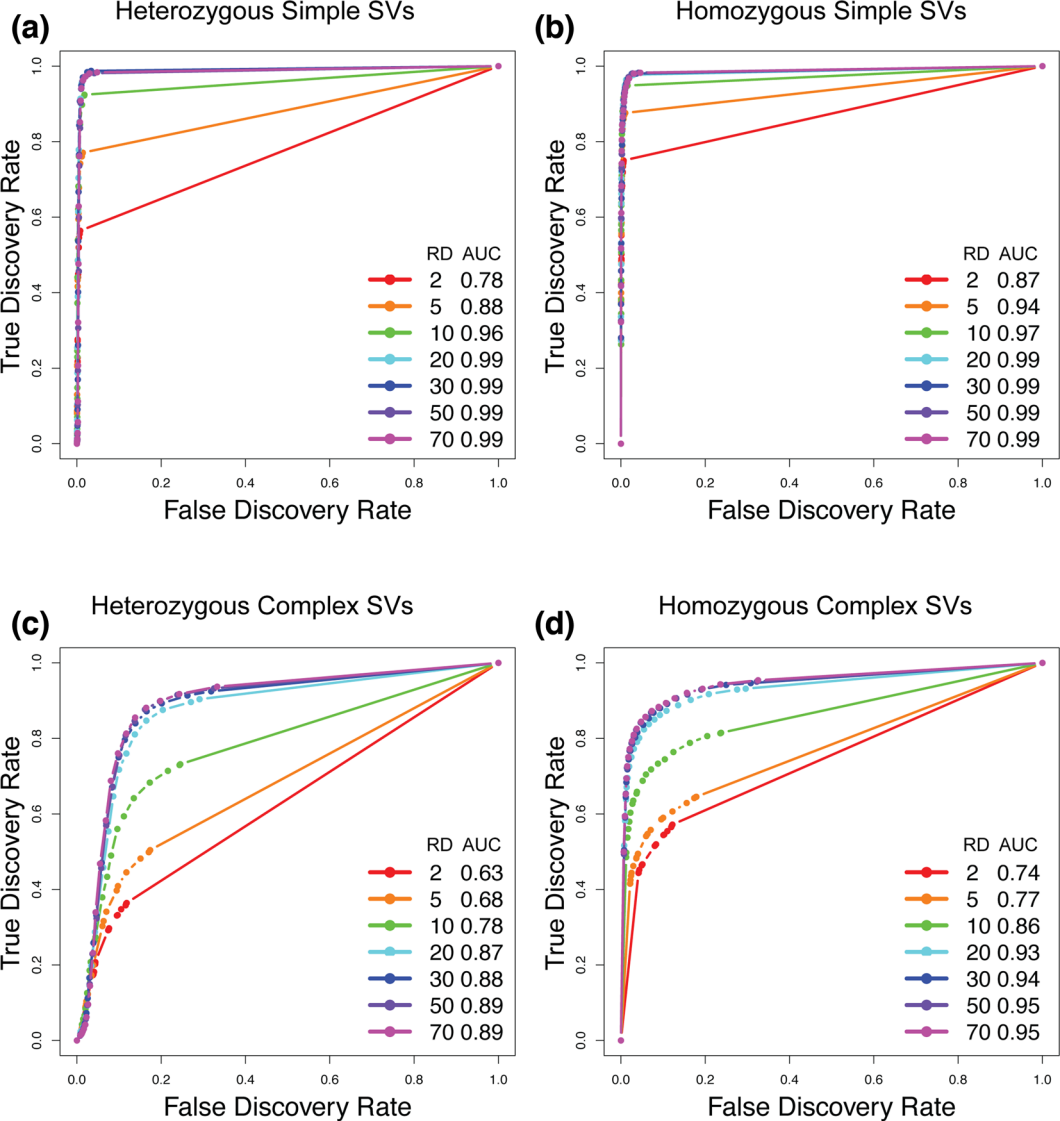
Accuracy of VaPoR on simulated heterozygous and homozygous SVs across different SV types. Receiver operator curves are shown for simple deletions, duplications, and inversions (**a, b**) as well as complex rearrangements including inverted duplications and deletion-inversion rearrangements (**c, d**). AUC: area under the curve; RD: read depth.

**Figure 4:**
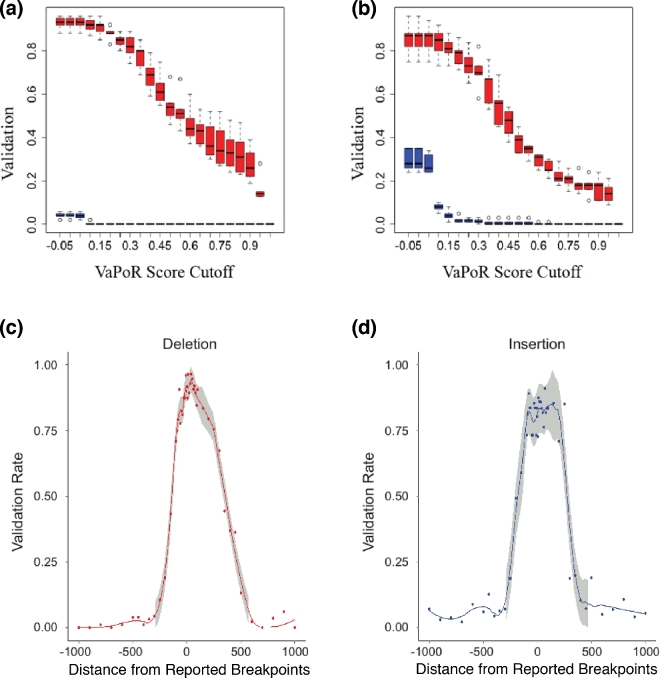
Validation rate and breakpoint accuracy of VaPoR on the 1000 Genomes Project phase 3 calls. VaPoR was applied on 5 individuals with reported SVs as a truth set: HG00513, HG00731, HG00732, NA19238, NA19239. The validation rates of deletions (**a**) and insertions (**b**) are shown here across different cutoff scores for VaPoR. Robustness to breakpoint accuracy was assessed by deviating breakpoints from their actual positions across varying distances for deletions (**c**) and insertions (**d**).

**Figure 5:**
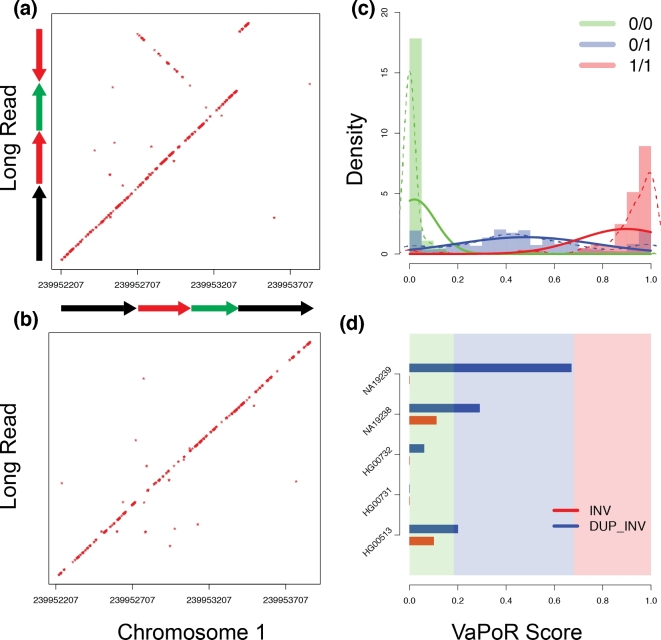
Validation and genotyping of assessed regions using VaPoR. (**a**) Dot plot of reference genome (GRCh38) to an aligned long read in NA19239 (m150208_160301_42225_c100732022550000001823141405141504_s1_p0/3831/0_12148) for a reported inversion at position chr1:239952707–239953529. The signature is consistent with an inverted duplication structure. (**b**) Dot plot of a different read (m150216_212941_42225_c100729442550000001823151505141565_s1_p0/106403/0_13205) against the same location, consistent with a non-variant (reference) structure. (**c**) Distribution of VaPoR scores on all reported SVs on chr1 in samples HG00513, HG00731, HG00732, NA19238, NA19239, stratified by color (solid) and modeled with a Gaussian mixture model (dashed). (**d**) VaPoR scores of SV above now stratified by color as indicated in (c) for both reported inversion (red) and predicted inverted duplication (blue).

#### Small variants assessment

For an SV *k* in a sample *s* that is covered by *n* reads, the recurrence matrix between each read and the reference sequences in original (*R_o_*) and altered (*R_a_*) format is calculated in the form of a dot plot. For each record *i* that corresponds to the fixed-size sequence window position and each format *R_x_* ε (*R_o_*, *R_a_*), we define a distance *d_i,k,s,Rx_* as the vertical distance between each record (X = *x_i,k,s,Rx_, Y = y_i,k,s,Rx_*) in matrix *x* and the diagonal (X = *x_i,k,s,Rx_, Y = x_i,k,s,Rx_*) such that *d_i,k,s,Rx_* = abs(*x_i,k,s,Rx__—_y_i,k,s,Rx_*), and the average distance of all records would be assigned as the score of each matrix:

\begin{equation*}
Scor{e_{k,s,Rx}} = \sum _{i = 1}^m {d_{i,k,s,Rx}} / m,
\end{equation*}

where *m* is the total number of records in the matrix. Sequences that share higher identity with the read will have a lower *Score_k,s,Rx_*, such that the score of each read is normalized as:

\begin{equation*}
Scor{e_{k,s,R}} = Scor{e_{k,s,{R_o}}}/ Scor{e_{k,s,{R_a}}} - 1,
\end{equation*}

where a positive *Score_k,s,R_* represents the superiority of the predicted structure versus the original and vice versa for negative *Score_k,s,R_*. There exists 1 exceptional case where a duplicated structure resides within the predicted SV such that the predicted structure would show higher *Score_k,s,R_* due to the multi-alignment of duplicated segments. To correct for these intrinsic duplications, VaPoR adopts the directed distance *d_i,k,s,Rx_ = *x*_i,k,s,Rx__—_*y*_i,k,s,Rx_* instead, and take the absolute value of their aggregation, such that the distances contributed by centrosymmetric duplicated segments would offset each other:

\begin{equation*}
Scor{e_{k,s,Rx}}^{'} = abs\left( \sum _{i = 1}^m {x_{i,k,s,Rx - }}{y_{i,k,s,Rx}}\right)/ m
\end{equation*}

#### Large variants assessment

For larger SVs where there are few, if any, long reads that can transverse the predicted SV, VaPoR assesses the quality of each predicted junction instead using:

\begin{equation*}
Scor{e_{k,s,Rx}} = \frac{{\mathop \sum \nolimits_{i = 1}^m I =
\left\{ {\begin{array}{@{}ll@{}}
1,& {\rm if}\ abs\left( {{x_{i,k,s,Rx}} - {y_{i,k,s,Rx}}} \right) < 0.15^{*}{x_{i,k,s,Rx}}\\
0,& {\rm otherwise} \end{array}} \right.}}{m} ,
\end{equation*}

where a larger *Score_k,s,Rx_* represents higher similarity between the read and the reference sequence. The normalized scores of each read are then defined as:

\begin{equation*}
Scor{e_{k,s,R}} = Scor{e_{k,s,{R_a}}}\ \ /\ Scor{e_{k,s,{R_o}}} - 1
\end{equation*}

***VaPoR score calculation***

With a score assigned to each read spanning through the predicted structural variants, the VaPoR score is summarized as:

\begin{equation*}
Scor{e_{k,s}} = \frac{{\sum\nolimits_{R = 1}^n I =
\left\{ {\begin{array}{@{}ll@{}}
1, & {\rm if}\ Scor{e_{k,s,R}} > 0\\
0, & {\rm otherwise} \end{array}}
\right.}}{n}
\end{equation*}

to represent the proportion of long reads supporting predicted structure.

The highest supportive score (max (*Score_k,s,R_*)) is also reported as a reference for users to meet the specific requirement of their study design, for which we recommend 0.1 as the cutoff.

#### Genotype assessment

The genotype and corresponding likelihood of a predicted SV are assessed by VaPoR using a method previously described for single nucleotide polymorphism genotyping [28]. Based on the assumption of 2 alleles per genomic site and *k* long reads adopted for the assessment, out of which *j* (***j*** ≤ ***k***) reads were assigned with a non-positive score, the log likelihood of a particular genotype *g* can be estimated as:

\begin{eqnarray*}
{{\boldsymbol{l}}_{\boldsymbol{g}}} &=& - {\boldsymbol{k*log}} (2) + \mathop \sum \limits_{{\boldsymbol{i}} = 1}^{\boldsymbol{j}} {\boldsymbol{log}}\left( {\left( {2 - {\boldsymbol{g}}} \right){{\boldsymbol{\varepsilon }}_{\boldsymbol{i}}} + {\boldsymbol{g}}\left( {1 - {{\boldsymbol{\varepsilon }}_{\boldsymbol{i}}}} \right)} \right) \nonumber\\
&&+\, \mathop \sum \limits_{{\boldsymbol{i}} = {\boldsymbol{j}} + 1}^{\boldsymbol{k}} {\boldsymbol{log}}
\left( {\left( {2 - {\boldsymbol{g}}} \right)
\left( {1 - {{\boldsymbol{\varepsilon }}_{\boldsymbol{i}}}} \right) + {\boldsymbol{g}}{{\boldsymbol{\varepsilon }}_{\boldsymbol{i}}}} \right)
\end{eqnarray*}

The error rate (ε_*i*_) was estimated as the proportion of negative reads across the homozygous alternative events and the positives across the homozygous reference, which are estimated to be 5% across the 1000 Genomes samples. The genotype with the highest likelihood is reported as the estimated genotype, with the second largest likelihood in –log10 normalized scale reported as the genotype quality score.

#### Flexible window size

By default, VaPoR uses a window size of 10 bp and requires an exact match between sequences, though these can be changed to user-defined parameters. However, many regions of the genome contain repetitive sequences, resulting in an abundance of spurious matches in the recurrence matrix, thus introducing bias to the assessment. To address this, VaPoR adopts a quality control step by iteratively assessing the reference sequence against itself and tabulating the proportion of matches along the diagonal. The window size initially starts at 10 bp and iteratively increases by 10 bp until either (i) the proportion of matches on the diagonal exceeds 40% and the current window size is kept or (ii) the window size exceeds 40 bp, whereby the event will be labeled non-assessable and excluded from the evaluation.

## Availability and requirements

Project name: VaPoR

Project home page: https://github.com/millslab/vapor

Operating systems: Linux, OS X

Programming languages: Python, R

Other requirements: Python v2.7.8+, rpy2, HTSeq, samtools v0.19+, pyfasta v0.5.2+, and pysam 0.9.1.4+.

An archival copy of the code on github, alignments, structural variants records, and other supplemental data are also available via the *GigaScience* repository, *Giga*DB [19].

## Additional files

Supplementary Figure 1: Sensitivity and FDR of validating heterozygously simulated structural variants were calculated at different cutoffs set for VaPoR score. Sensitivity and FDR both decrease with the cutoff increasing, with >90% sensitivity and <10% FDR achieved at cutoff = 0.1.

Supplementary Figure 2: Sensitivity and FDR of validating homozygously simulated structural variants were calculated at different cutoffs set for VaPoR score. Sensitivity and FDR both decrease with the cutoff increasing, with >90% sensitivity and <10% FDR achieved at cutoff = 0.1 – 0.25.

Supplementary Figure 3: Length distribution of PacBio reads in HG00513 (red), and the corresponding distribution of SVs reported in the same sample (blue). The median length of aligned PacBio long reads is 15.6 Kb, and ∼5% of the 1 KGP phase 3 predictions in HG00513 have lengths over the median.

Supplementary Figure 4: An example of VaPoR on large SVs predictions that have few long sequences fully transverse. (a) The IGV screenshot of the region on chr1: 72300660-72346132, which indicates a homozygous deletion of 45.5 Kb. (b) The recurrence plots of pacbio read (read name: m150923_001907_42216_c100828312550000001823180911251591_s1_p0/81227/27314_30304) versus reference sequence in the original and altered format.

Supplementary Figure 5: Plot of validation rate when validating the simulated SVs with fake breakpoints deviated from the real ones by different bases. Validation rates are averaged from simulated deletion, insertion, inversion, and tandem duplication at ×30 coverage.

Supplementary Figure 6: Plot of validation rate when validating the simulated SVs with fake breakpoints deviated from the real ones by different bases. Validation rates are shown for simulated deletion, insertion, inversion, and tandem duplication at ×30 coverage.

Supplementary Figure 7: Averaged run time (seconds) of each simulated SV summarized and plotted at different read depths. Simple and complex SVs are estimated separately, shown in red and blue lines, respectively.

## Competing interests

None declared.

## Funding

This work was supported by the National Institutes of Health (R01HG007068). A.M.W. was supported by the Genome Science Training Program at the University of Michigan (5T32HG000040).

## Author contributions

X.Z. designed the algorithm, wrote the program, comparatively benchmarked the different algorithms, and wrote the manuscript. A.M.W. generated simulated data, aided in assessment testing, and revised the manuscript. R.E.M. conceived the study, modified the algorithm, and revised the manuscript. All authors read and approved the final manuscript.

## Supplementary Material

GIGA-D-17-00068_Original-Submission.pdf

GIGA-D-17-00068_Revision-1.pdf

GIGA-D-17-00068_Revision-2.pdf

Response-to-Reviewer-Comments_Original-Submission.pdf

Response-to-Reviewer-Comments_Revision-1.pdf

Reviewer-1-Report-(Original-Submission).pdf

Reviewer-1-Report-(Revision-1).pdf

Reviewer-2-Report-(Original-Submission).pdf

Reviewer-2-Report-(Revision-1).pdf

Supplement Materials

## References

[1] Brand H, Pillalamarri V, Collins RL Cryptic and complex chromosomal aberrations in early-onset neuropsychiatric disorders. Am J Hum Genet 2014;95(4):454–61. 25279985 10.1016/j.ajhg.2014.09.005 PMC4185111

[2] Chiang C, Jacobsen JC, Ernst C Complex reorganization and predominant non-homologous repair following chromosomal breakage in karyotypically balanced germline rearrangements and transgenic integration. Nat Genet 2012;44(4):390–7. 22388000 10.1038/ng.2202 PMC3340016

[3] Layer RM, Chiang C, Quinlan AR LUMPY: a probabilistic framework for structural variant discovery. Genome Biol 2014;15(6):R84. 24970577 10.1186/gb-2014-15-6-r84 PMC4197822

[4] Rausch T, Zichner T, Schlattl A DELLY: structural variant discovery by integrated paired-end and split-read analysis. Bioinformatics 2012;28(18):i333–9. 22962449 10.1093/bioinformatics/bts378 PMC3436805

[5] Zhao X, Emery SB, Myers B Resolving complex structural genomic rearrangements using a randomized approach. Genome Biol 2016; doi:10.1186/s13059-016-0993-1. 10.1186/s13059-016-0993-1 PMC4901421 27287201

[6] Chong Z, Ruan J, Gao M novoBreak: local assembly for breakpoint detection in cancer genomes. Nat Meth 2017;14(1):65–67. 10.1038/nmeth.4084 PMC5199621 27892959

[7] Rhoads A, Au KF PacBio sequencing and its applications. Genomics Proteomics Bioinformatics 2015;13(5):278–89. 26542840 10.1016/j.gpb.2015.08.002 PMC4678779

[8] Travers KJ, Chin C-S, Rank DR A flexible and efficient template format for circular consensus sequencing and SNP detection. Nucleic Acids Res 2010;38(15):e159. 20571086 10.1093/nar/gkq543 PMC2926623

[9] Chaisson MJP, Huddleston J, Dennis MY Resolving the complexity of the human genome using single-molecule sequencing. Nature 2015;517(7536):608–11. 25383537 10.1038/nature13907 PMC4317254

[10] Pendleton M, Sebra R, Pang AWC Assembly and diploid architecture of an individual human genome via single-molecule technologies. Nat Methods 2015; doi:10.1038/nmeth.3454. 10.1038/nmeth.3454 PMC4646949 26121404

[11] Shi L, Guo Y, Dong C Long-read sequencing and de novo assembly of a Chinese genome. Nat Commun 2016;7:12065. 27356984 10.1038/ncomms12065 PMC4931320

[12] Koren S, Schatz MC, Walenz BP Hybrid error correction and de novo assembly of single-molecule sequencing reads. Nat Biotechnol 2012;30(7):693–700. 22750884 10.1038/nbt.2280 PMC3707490

[13] Huddleston J, Chaisson MJ, Meltz Steinberg K Discovery and genotyping of structural variation from long-read haploid genome sequence data. Genome Res 2016; doi:10.1101/gr.214007.116. 10.1101/gr.214007.116 PMC5411763 27895111

[14] Carvalho AB, Dupim EG, Goldstein G Improved assembly of noisy long reads by k-mer validation. Genome Res 2016;26(12):1710–20. 27831497 10.1101/gr.209247.116 PMC5131822

[15] Gibbs AJ, Mcintyre GA The diagram, a method for comparing sequences. Its use with amino acid and nucleotide sequences. Eur J Biochem 1970;16(1):1–11. 5456129 10.1111/j.1432-1033.1970.tb01046.x

[16] Dunham I, Kundaje A, Aldred SF An integrated encyclopedia of DNA elements in the human genome. Nature 2012;489(7414):57–74. 22955616 10.1038/nature11247 PMC3439153

[17] Ono Y, Asai K, Hamada M PBSIM: PacBio reads simulator—toward accurate genome assembly. Bioinformatics 2013;29(1):119–21. 23129296 10.1093/bioinformatics/bts649

[18] University of Michigan https://umich.box.com/v/vapor (8 July 2017, date last accessed).

[19] Zhao X, Weber AM, Mills RE Supporting data for “A recurrence based approach for validating structural variation using long-read sequencing technology.” GigaScience Database 2017 http://dx.doi.org/10.5524/100325. 10.1093/gigascience/gix061 PMC5737365 28873962

[20] Sudmant PH, Rausch T, Gardner EJ An integrated map of structural variation in 2,504 human genomes. Nature 2015;526(7571):75–81. 26432246 10.1038/nature15394 PMC4617611

[21] NCBI, 1000 Genomes Project ftp://ftp-trace.ncbi.nih.gov/1000genomes/ftp/phase3/integrated_sv_map/ (8 July 2017, date last accessed).

[22] EBI, 1000 Genomes Project http://ftp.1000genomes.ebi.ac.uk/vol1/ftp/data_collections/hgsv_sv_discovery/ (8 July 2017, date last accessed).

[23] Hall Lab GitHub Repository https://github.com/hall-lab/long-read-validation (8 July 2017, date last accessed).

[24] EBI, 1000 Genomes Project http://ftp.1000genomes.ebi.ac.uk/vol1/ftp/technical/working/20131209_na12878_pacbio/si/ (8 July 2017, date last accessed).

[25] EBI, 1000 Genomes Project http://ftp.1000genomes.ebi.ac.uk/vol1/ftp/phase3/integrated_sv_map/supporting/NA12878/moleculo/alignment/ (8 July 2017, date last accessed).

[26] Auton A, Abecasis GR, Altshuler DM A global reference for human genetic variation. Nature 2015;526(7571):68–74. 26432245 10.1038/nature15393 PMC4750478

[27] Robinson JT, Thorvaldsdottir H, Winckler W Integrative genomics viewer. Nat Biotechnol 2011;29(1):24–26. 21221095 10.1038/nbt.1754 PMC3346182

[28] Li H A statistical framework for SNP calling, mutation discovery, association mapping and population genetical parameter estimation from sequencing data. Bioinformatics 2011;27(21):2987–93. 21903627 10.1093/bioinformatics/btr509 PMC3198575

