# Supplementary material for: A recurrence-based approach for validating structural variation using long-read sequencing technology
Source: Gigascience. 2017 Jul 19;6(8):1–9. doi: 10.1093/gigascience/gix061 (PMC5737365; doi:10.1093/gigascience/gix061)
Supplement: GIGA-D-17-00068_Original-Submission.pdf [file gix061_giga-d-17-00068_original-submission.pdf]

Manuscript Click here to download Manuscript manuscript.docx

A recurrence based approach for validating structural variation using long-read sequencing technology.

Xuefang Zhao[1], Alexandra M. Weber[1], and Ryan E. Mills[1,2,#]

[1]Department of Computational Medicine and Bioinformatics, University of Michigan, Ann Arbor, MI, 48109, USA, [2]Department of Neurology, University of Michigan, Ann Arbor, MI, 48109, USA, [3]Veterans Affairs Medical Center, Ann Arbor, MI, 48105, USA, [4]Department of Human Genetics, University of Michigan, Ann Arbor, MI, 48109, USA

[#]Corresponding author: remills@umich.edu

ABSTRACT

Background

Although numerous algorithms have been developed to identify structural variation (SVs) in genomic sequences, there is a dearth of approaches that can be used to evaluate their results. This is significant, as the accurate identification of structural variation is still an outstanding but important problem in genomics. The emergence of new sequencing technologies that generate longer sequence reads can, in theory, provide direct evidence for all types of SVs regardless of the length of region through which it spans. However, current efforts to use these data in this manner require the use of large computational resources to assemble these sequences as well as visual inspection of each region.

Results

Here we present VaPoR, a highly efficient algorithm that autonomously validates large SV sets using long read sequencing data. We assessed of the performance of VaPoR on SVs in both simulated and real genomes and report a high-fidelity rate for overall accuracy across different levels of sequence depths. We show that VaPoR can interrogate a much larger range of SVs while still matching existing methods in terms of false positive validations and providing additional features considering breakpoint precision and predicted genotype. We further show that VaPoR can run quickly and efficiency without requiring a large processing or assembly pipeline.

Conclusions

VaPoR serves as a high efficient long read based validation approach for genomic SVs that requires relatively low read depth and computing resources and thus will provide utility with targeted or low-pass sequencing coverage for accurate SV assessment.

Keywords: structural variation, copy number variation, sequence analysis

BACKGROUND

Structural variants (SVs) are one of the major forms of genetic variation in humans and have been revealed to play important roles in numerous diseases including cancers and neurological disorders [1, 2]. Various approaches have been developed and applied to paired-end sequencing to detect SVs in whole genomes [3-6], however individual algorithms often exhibit complementary strengths that sometimes lead to disagreements as to the precise structure of the underlying variant. The emergence of long read sequencing technology, such as Single Molecule Real-Time (SMRT) sequencing from Pacific Biosciences (PacBio) [7, 8], can deliver reads ranging from several to hundreds of kilobases and provide direct evidence for the presence of an SV. Current strategies make use of de novo assembly to create long contigs with minimized error rate [9-11], and then predict SVs, usually with single base resolution, through direct comparison of the assembly against the reference. Though such approaches are powerful, they require both a very high sequencing depth and significant computing power and are currently impracticable for many ongoing research studies.

The additional information obtained from using long reads can still be leveraged to improve variant calling, however. Indeed, such approaches have already been implemented to combine high depth Illumina sequencing with lower depth PacBio reads to improve error correction and variant calling in the context of *de novo* genome assembly [12]. With structural variation, the current state of the art is to use long reads to manually assess potential SVs using subsequent recurrence (dot) plots [13], where the sequences are compared against the reference through a fixed size sliding window (k-mer) and the matches are plotted for visual inspection. The k-mer method is of higher robustness compared against the direct sequences comparison [14], which is why these types of dot plots have been used for decades to examine the specific features of sequence alignments [15]. However, they require manual curation and, coupled with the computational costs of sequence assembly, are time-consuming and inefficient at scale for the high throughput validation of large sets of SVs.

Here, we present a high-speed long read based assessment tool, VaPoR, that scores each SV prediction by autonomously analyzing the recurrence of windows within a local read against the reference genome in both their original and rearranged format per the prediction. A positive score of each read on the altered reference, normalized against the score of the read on the original reference, supports the predicted structure. A baseline model is constructed as well by interrogating the reference sequence

against itself at the query location. We show that our approach can quickly and accurately distinguish true from false positive predictions of both simple and complex SVs as well as their underlying genotypes and is also able to assess the breakpoint accuracy of individual algorithms.

DATA DESCRIPTION

Simulated Data:

Non-overlapping simple deletions, inversions, insertions and duplications as well as complex structural variants as previously categorized [5] were independently incorporated into GRCh38 in both heterozygous and homozygous states, excluding regions of the genome that are known to be difficult to assess as described from the ENCODE project [16]. Detailed descriptions of each simulated SV types simulated are summarized in Supplementary Tables 1- 2. We applied PBSIM [17] to simulate the modified reference sequences to different read depth ranging from 2X to 70X with a parameters difference-ratio of 5:75:20, length-mean 12000, accuracy-mean 0.85 and *model_qc model_qc_clr*. Simulated data can be obtained from https://umich.box.com/v/vapor.

Real Data

We applied VaPoR to a set of diverse samples (HG00513 from CHS, HG00731 and HG00732 from PUR, NA19238 and NA19239 from YRI) that were initially sequenced by the 1000 Genomes Project and for which a high-quality set of SVs were reported in the final phase of the project [18]. These samples were recently re-sequenced using PacBio and therefore provides a platform for assessing VaPoR on known data. The 1000 Genomes Project (1KGP) Phase 3 data were obtained from ftp://ftp-trace.ncbi.nih.gov/1000genomes/ftp/phase3/integrated_sv_map/ and lifted over to GRCh38. PacBio sequence data were obtained from http://ftp.1000genomes.ebi.ac.uk/vol1/ftp/data_collections/hgsv_sv_discovery/.

We have also compared VaPoR against the long read validation approach developed by Layer et al. [3], which requires both PacBio and Moleculo long sequences for full evaluation of SVs. These comparisons made use of NA12878, one of few samples that have been sequenced with various technologies

including Illumina NGS, PacBio and Moleculo with a truth SV set included in the 1KGP Phase 3 report. The software for the long-read validation approach was obtained from: https://github.com/hall-lab/long-read-validation. The PacBio and the Moleculo sequences of this individual were obtained from : http://ftp.1000genomes.ebi.ac.uk/vol1/ftp/technical/working/20131209_na12878_pacbio/si/ and http://ftp.1000genomes.ebi.ac.uk/vol1/ftp/phase3/integrated_sv_map/supporting/NA12878/moleculo/alignment/ respectively.

RESULTS

We assessed the performance of VaPoR on both simulated sequences and real genomes from the 1000 Genomes Project to assess the following characteristics: sensitivity and false discovery rate on validating structural variants in simple and complex structures; sensitivity of VaPoR on validating different levels of predicted breakpoint efficacy; stratification of VaPoR scores by genotype; and time and computational cost of VaPoR.

VaPoR on Simulated Data

We applied VaPoR to simulated simple deletions, inversions, insertions and duplications as well as complex structural variants and first assessed the proportion of SVs that VaPoR is capable of interrogating (i.e. passed VaPoR QC). We found that VaPoR can successfully evaluate >80% of insertions, >85% deletion-duplications and >90% SVs in all other categories when the read depth is 10X or higher. We then assessed the sensitivity and false discovery rate (FDR) at different VaPoR score cutoffs and found that a sensitivity >90% is achieved for most SV types across a wide range of read depths while maintaining a false discovery rate <10% at a VaPoR score cutoff of 0.15 (Supplemental Figures 1-2). We further observed that there were no significant changes of sensitivity or false discovery rate once the read depth was at or above 20X and is consistent across different SV types (Figure 2, Supplemental Figure 3-4, Supplemental Table 3).

VaPoR on 1000 Genomes Project Samples

We next examined SVs reported on chr1 of 5 individuals from the 1000 Genomes Project [19] to assess the sensitivity of VaPoR on real genomes (Table 1). We first observed that >95% of deletions and insertions could be successfully evaluated by VaPoR. For inversions, there were a limited number of events reported but at maximum only 1 event failed the VaPoR quality control per individual. A sensitivity of >90% was achieved for deletions (Figure 3a) and >80% for insertions (Figure 3b) at the recommended cutoff of 0.15. To examine the false validation rate of VaPoR, we modified reported events on chr2 to appear at the same coordinates on chr1 and assessed them as though they were real events using the same sequence data set. VaPoR validated very few deletions or inversion and <10% of insertions. We further assessed the performance of VaPoR to validate SVs with varying degrees of breakpoint accuracy. Real coordinates were artificially shifted each direction by -1000 to 1000 base pairs and re-assessed with VaPoR for both simulated and real samples. In both cases, VaPoR exhibited a robust validation score up to approximately 200bp overall, with some slight differences observed between different SV types (Figure 3c,d).

We also compared VaPoR against a long-read validation approach developed in conjunction with Lumpy [3] using SVs on chr1 of NA12878 reported by the 1000 Genomes Project Phase 3. VaPoR achieved a sensitivity of 72% for deletions and 86% for insertions, while the Lumpy-associated approach was only able to assess 11% and 0% respectively. Both approaches exhibited a very low false validation rate when synthetically assigning the variants to chr2, with 0 for all SV types by the Layer et al approach and varying between 0 and 2.5% for VaPoR (Supplemental Table 4).

Discrimination of SV types and genotypes

We identified a small number of SVs in the high quality 1000 Genomes set that did not validate with VaPoR. Previous studies have shown that complex rearrangements are often misclassified as simple structural changes [5, 13], and indeed upon manual inspection these appeared to consist of multiple connected rearrangements. For example, we observed a reported inversion in HG00513 and NA19239 on chromosome 1 (chr1:239952707-239953529) that was invalidated by VaPoR; an investigation into the long-reads aligned in the region showed the signature of an inverted duplication (Figure 4a) which, when incorporated into a modified reference that location, matched almost exactly with the read sequence (Figure 4b).

We further explored the distribution of VaPoR scores for this region and others across the sample set and observed clear delineations between allelic copy number when fitted with a Gaussian mixture model allowing for the generation of genotype likelihoods for each site (Figure 4c). These tracked with our expected genotypes for the inverted duplication on chr1 across the 5 individuals queried while showing no support for the originally predicted inversion (Figure 4d). This shows that VaPoR is not only able to accurately genotype variants but can also distinguish between similar but distinct SV predictions in the same region.

Runtime and efficiency

The computation runtime of VaPoR was assessed using 2 Intel Xeon Intel Xeon E7-4860 processors with 4GB RAM each on both simulated and real genomes. The runtime of simulated event was observed to increase linearly with read depth (Supplemental Figure 7). For events sequenced up to 20X, VaPoR takes ~3 seconds to assess a simple SV and ~5s for a complex event. The assessment of real samples sequenced at 20X required ~1.4 seconds to assess a simple deletion or insertion and ~6 seconds for an inversion (Supplemental Table 5).

DISCUSSION

Here we present an automated assessment approach, named VaPoR, for exploring various features of predicted genomic structural variants using long read sequencing data. VaPoR directly compares the input reads with the reference sequences with relatively straightforward computational metrics, thus achieving high efficiency in both run time and computing cost. VaPoR exhibits high sensitivity and specificity in both simulated and real genomes, with the capability of discriminating partially resolved SVs either consisting of similar but incorrect SV types at the same location or correct SVs with offset breakpoints. Furthermore, we show that VaPoR performs well at low read depths (5-10X), thus providing the option of systematically assessing large-scale SVs with a lower sequencing cost.

METHODS

VaPoR Workflow

VaPoR takes in aligned sequence reads in BAM format and predicted SVs (>50bp) in various formats including VCF and BED. Evaluation of an SV is performed by comparing long reads that go through the event against reference sequences in two formats: (a) the original human reference to which the sample is aligned and (b) a modified reference sequence altered to match the predicted structural rearrangement. A recurrence matrix is then derived by sliding a fixed-size window with 1bp step through each read to mark positions where the read sequence and reference are identical. The matching patterns are then assessed as to the validity of the SV as described below and a validation score is reported. Given the large variance of SVs lengths, each SV is stratified into one of two groups: smaller SVs that can be completely encompassed within multiple (>10 by default) long sequences and larger events that are rarely covered by individual long reads, with different statistical model applied. The VaPoR workflow is briefly summarized in Figure 1.

Small Variants Assessment:

For an SV k in sample s that is covered by n reads, the recurrence matrix between each read and the reference sequences in original (R_o) and altered (R_a) format is calculated. The vertical distance between each record ($x_{i,k,s,Rx}$, $y_{i,k,s,Rx}$) in matrix x and the diagonal ($x_{i,k,s,Rx}$, $x_{i,k,s,Rx}$) line is calculated as $d_{i,k,s,Rx}$ = abs($x_{i,k,s,Rx}$ - $y_{i,k,s,Rx}$), and the average distance of all records would be exported as the score of each matrix:

$$Score_{k,s,Rx} = \sum_{i=1}^{m} d_{i,k,s,Rx} \, / \, m,$$

where m is the total number of records in the matrix. Sequences that share higher identity with the read shall have a lower $Score_{k,s,Rx}$, such that the score of each read is normalized as:

$$Score_{k,s,R} = Score_{k,s,R_o} \, / \, Score_{k,s,R_a} - 1,$$

where a positive $Score_{k,s,R}$ represents the superiority of the predicted structure versus the original and vice versa for negative $Score_{k,s,R}$, with one exceptional case where there exists a duplicated structure in the predicted SV such that the predicted structure would show higher $Score_{k,s,R}$ due to the multi-alignment of duplicated segments. To correct for duplications, VaPoR adopts the directed distance $d_{i,k,s,Rx} = x_{i,k,s,Rx} - y_{i,k,s,Rx}$ instead such that the distance contributed by centrosymmetric duplicated segments would offset each other.

Large Variants Assessment:

For larger SVs where there are few, if any, long reads that can transverse the predicted SV, VaPoR assesses the quality of each predicted junction instead using:

$$Score_{k,s,Rx} = \frac{\sum_{i=1}^{m} I = \begin{cases} 1, if\ abs\left(x_{i,k,s,Rx} - y_{i,k,s,Rx}\right) < 0.15 * x_{i,k,s,Rx} \\ 0, otherwise \end{cases}}{m},$$

where a larger $Score_{k,s,Rx}$ represents higher similarity between the read and the reference sequence. The normalized scores of each read is then defined as:

$$Score_{k,s,R} = Score_{k,s,R_a} \,/\, Score_{k,s,R_o} - 1,$$

VaPoR Score Calculation:

With a score assigned to each read spanning through the predicted structural variants, the VaPoR score is summarized as:

$$Score_{k,s} = \frac{\sum_{R=1}^{n} I = \begin{cases} 1, if\ \ Score_{k,s,R} > 0 \\ 0, otherwise \end{cases}}{n}$$

to represent the proportion of long reads supporting predicted structure.

The highest supportive score (max($Score_{k,s,R}$)) is also reported as a reference for users to meet the specific requirement of their study design, for which we recommend 0.1 as the cutoff.

Flexible window size:

By default, VaPoR uses a window size of 10bp and requires an exact match between sequences, though these can be changed to user-defined parameters. However, many regions of the genome contain repetitive sequences resulting in an abundance of spurious matches in the recurrence matrix, thus introducing bias to the assessment. To address this, VaPoR adopts a quality control step by iteratively assessing the reference sequence against itself and tabulating the proportion of matches along the diagonal. The window size initially starts at 10bp and iteratively increases by 10bp until either (a) the proportion of matches on the diagonal exceeds 40% and the current window size is kept or (b) the window size exceeds 40bp whereby the event will be labeled as ‘non-assessable and excluded from the evaluation.

FIGURE LEGENDS

Figure 1. Flowchart describing the VaPoR algorithm. As input, the algorithm requires a set of structural variants in either VCF or BED format, a series of long reads and/or sequence contigs in BAM format, and the corresponding reference sequence. VaPoR then interrogates each variant individually at its corresponding reference location, assesses the quality of the region and assigns a score.

Figure 2. Accuracy of VaPoR on simulated heterozygous and homozygous SVs at varying degrees of sequence coverage and VaPoR score cut-offs. Receiver operator curves (ROC) are shown for simple deletions, duplications and inversions (a,b) as well as complex rearrangements including inverted duplications and deletion-inversion rearrangements (c,d).

Figure 3. Validation rate and breakpoint accuracy of VaPoR on the 1000 Genomes Projects phase 3 calls. VaPoR was applied on 5 individuals with reported SVs as a truth set: HG00513, HG00731, HG00732, NA19238, NA19239. The validation rate of deletions (a) and insertions (b) are shown here across different cutoff scores for VaPoR. Robustness to breakpoint accuracy was assessed by deviating breakpoints from their actual positions across varying distances for deletions (c) and insertions (d).

Figure 4. Validation and genotyping of assessed regions using VaPoR. (a) Recurrence plot of reference genome (GRCh38) to an aligned long read in NA19239 (m150208_160301_42225_c100732022550000001823141405141504_s1_p0/3831/0_12148) for a reported inversion at position chr1:239952707-239953529. The signature is consistent with an inverted duplication structure. (b) Recurrence plot of a different read (m150216_212941_42225_c100729442550000001823151505141565_s1_p0/106403/0_13205) against the same location, consistent with a non-variant (reference) structure. (c) Distribution of VaPoR scores on all reported SVs on chr1 in samples HG00513, HG00731, HG00732, NA19238, NA19239, stratified by color (solid) and modeled with a Gaussian mixture model (dashed). (d) VaPoR scores of SV above now stratified by color as indicated in (c) for both reported inversion (red) and predicted inverted duplication (blue).

TABLES

Table 1. Sensitivity and false discovery rate of different SV types

	deletion	insertion	inversion
Sample	Sens/FDR	Sens/FDR	Sens/FDR
HG00513	0.96/0.00 (0.94[1])	0.80/0.05 (0.93)	0.50/0.00 (0.71)
HG00731	0.94/0.00 (0.96)	0.85/0.07 (0.97)	0.60/0.00 (1.00)
HG00732	0.92/0.00 (0.98)	0.92/0.08 (0.96)	0.33/0.00 (0.86)
NA19238	0.90/0.00 (0.93)	0.88/0.10 (0.96)	1.00/0.00 (1.00)
NA19239	0.87/0.02 (0.95)	0.73/0.09 (0.96)	0.33/0.00 (1.00)

[1]Proportion of SVs passed VaPoR QC, as listed in brackets, are counted for events on chr1 and chr2 together.

Availability and Requirements

Project name: VaPoR

Project home page: https://github.com/millslab/vapor

Operating systems: Linux, OS X

Programming languages: Python, R

Other requirements: Python v2.7.8+, rpy2, HTSeq, samtools v0.19+, pyfasta v0.5.2+, and pysam 0.9.1.4+.

Acknowledgements

We thank the Human Genome Structural Variation Consortium (HGSVC) for generating and providing the deep PacBio sequencing. We also thank Yuanfang Guan and Kerby Shedden for discussions over specific statistical considerations.

Funding

This work was supported by the National Institutes of Health [R01HG007068]. AMW was supported by the Genome Science Training Program at the University of Michigan [5T32HG000040]

Authors' contributions

XZ designed the algorithm, wrote the program, comparatively benchmarked the different algorithms, and wrote the manuscript. AMW generated simulated data, aided in assessment testing, and revised the manuscript. REM conceived the study, modified the algorithm, and revised the manuscript. All authors read and approved the final manuscript.

Competing interests

None declared.

REFERENCES

1. Brand H, Pillalamarri V, Collins RL, Eggert S, O'Dushlaine C, Braaten EB, et al. Cryptic and complex chromosomal aberrations in early-onset neuropsychiatric disorders. Am J Hum Genet. 2014;95 4:454-61. doi:10.1016/j.ajhg.2014.09.005.
2. Chiang C, Jacobsen JC, Ernst C, Hanscom C, Heilbut A, Blumenthal I, et al. Complex reorganization and predominant non-homologous repair following chromosomal breakage in karyotypically balanced germline rearrangements and transgenic integration. Nature Genetics. 2012;44 4:390-U195. doi:10.1038/ng.2202.
3. Layer RM, Chiang C, Quinlan AR and Hall IM. LUMPY: a probabilistic framework for structural variant discovery. Genome Biol. 2014;15 6:R84. doi:10.1186/gb-2014-15-6-r84.
4. Rausch T, Zichner T, Schlattl A, Stutz AM, Benes V and Korbel JO. DELLY: structural variant discovery by integrated paired-end and split-read analysis. Bioinformatics. 2012;28 18:i333-i9. doi:10.1093/bioinformatics/bts378
bts378 [pii].
5. Zhao X, Emery SB, Myers B, Kidd JM and Mills RE. Resolving complex structural genomic rearrangements using a randomized approach. Genome Biology. 2016;17 doi:10.1186/s13059-016-0993-1.
6. Chong Z, Ruan J, Gao M, Zhou W, Chen T, Fan X, et al. novoBreak: local assembly for breakpoint detection in cancer genomes. Nat Methods. 2017;14 1:65-7. doi:10.1038/nmeth.4084.
7. Rhoads A and Au KF. PacBio Sequencing and Its Applications. Genomics Proteomics Bioinformatics. 2015;13 5:278-89. doi:10.1016/j.gpb.2015.08.002.
8. Travers KJ, Chin CS, Rank DR, Eid JS and Turner SW. A flexible and efficient template format for circular consensus sequencing and SNP detection. Nucleic Acids Res. 2010;38 15:e159. doi:10.1093/nar/gkq543.
9. Chaisson MJ, Huddleston J, Dennis MY, Sudmant PH, Malig M, Hormozdiari F, et al. Resolving the complexity of the human genome using single-molecule sequencing. Nature. 2015;517 7536:608-11. doi:10.1038/nature13907.
10. Pendleton M, Sebra R, Pang AW, Ummat A, Franzen O, Rausch T, et al. Assembly and diploid architecture of an individual human genome via single-molecule technologies. Nat Methods. 2015; doi:10.1038/nmeth.3454.
11. Shi L, Guo Y, Dong C, Huddleston J, Yang H, Han X, et al. Long-read sequencing and de novo assembly of a Chinese genome. Nature communications. 2016;7:12065. doi:10.1038/ncomms12065.
12. Koren S, Schatz MC, Walenz BP, Martin J, Howard JT, Ganapathy G, et al. Hybrid error correction and de novo assembly of single-molecule sequencing reads. Nat Biotechnol. 2012;30 7:693-700. doi:10.1038/nbt.2280.
13. Huddleston J, Chaisson MJ, Meltz Steinberg K, Warren W, Hoekzema K, Gordon DS, et al. Discovery and genotyping of structural variation from long-read haploid genome sequence data. Genome Res. 2016; doi:10.1101/gr.214007.116.
14. Carvalho AB, Dupim EG and Goldstein G. Improved assembly of noisy long reads by k-mer validation. Genome Res. 2016;26 12:1710-20. doi:10.1101/gr.209247.116.
15. Gibbs AJ and McIntyre GA. The diagram, a method for comparing sequences. Its use with amino acid and nucleotide sequences. European journal of biochemistry. 1970;16 1:1-11.

16. Consortium EP. An integrated encyclopedia of DNA elements in the human genome. Nature. 2012;489 7414:57-74. doi:10.1038/nature11247.
17. Ono Y, Asai K and Hamada M. PBSIM: PacBio reads simulator--toward accurate genome assembly. Bioinformatics. 2013;29 1:119-21. doi:10.1093/bioinformatics/bts649.
18. Sudmant PH, Rausch T, Gardner EJ, Handsaker RE, Abyzov A, Huddleston J, et al. An integrated map of structural variation in 2,504 human genomes. Nature. 2015;526 7571:75-+. doi:10.1038/nature15394.
19. Genomes Project C, Auton A, Brooks LD, Durbin RM, Garrison EP, Kang HM, et al. A global reference for human genetic variation. Nature. 2015;526 7571:68-74. doi:10.1038/nature15393.

Figure 1

Click here to download Figure figure1.png

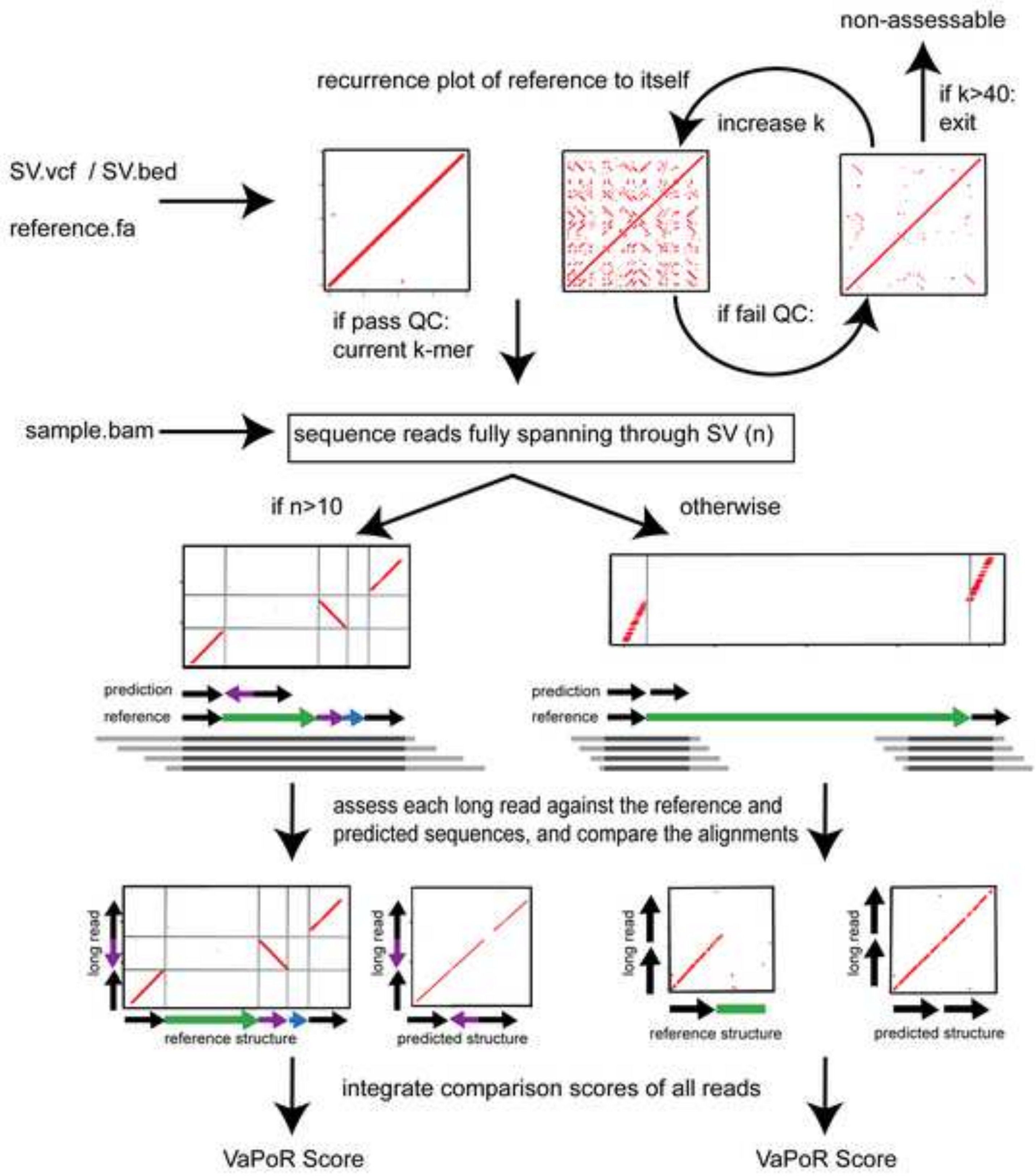

Figure 2

Click here to download Figure figure2.png

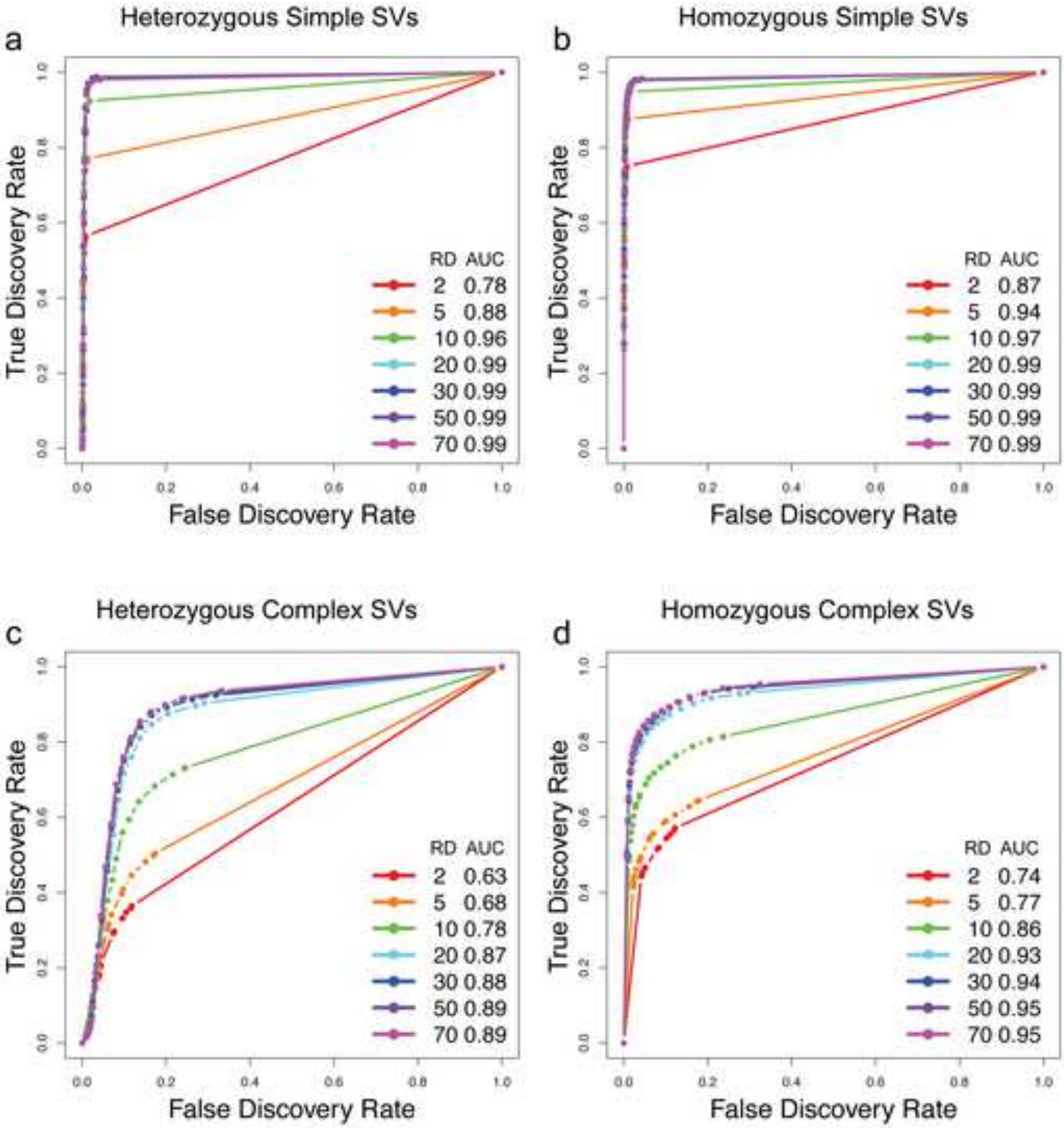

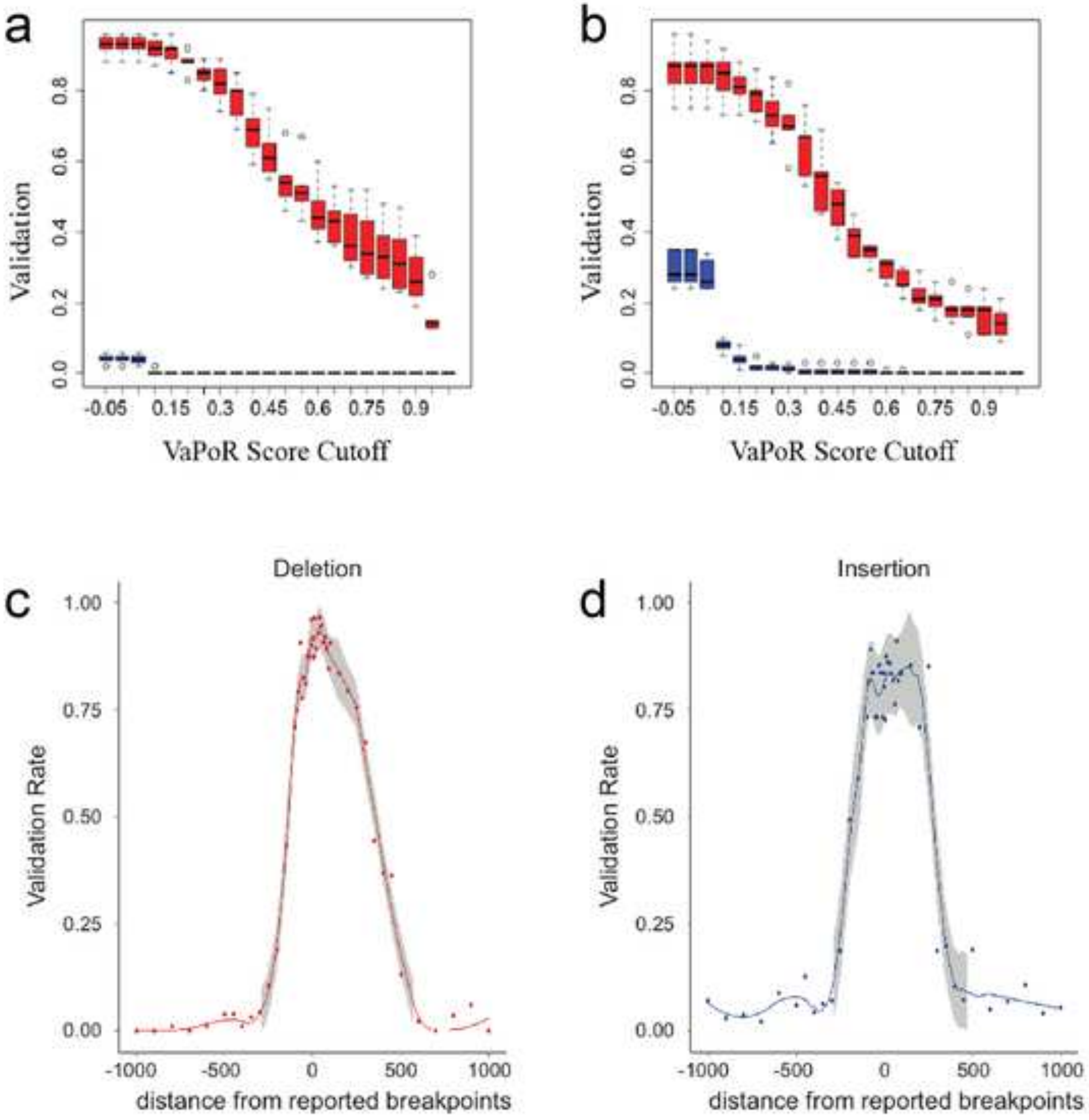
a
b
Validation
0.0
0.2
0.4
0.6
0.8
-0.05
0.15
0.3
0.45
0.6
0.75
0.9
VaPoR Score Cutoff
c
d
Deletion
Insertion
Validation Rate
0.00
0.25
0.50
0.75
1.00
-1000
-500
0
500
1000
distance from reported breakpoints

Figure 4

Click here to download Figure figure4.png

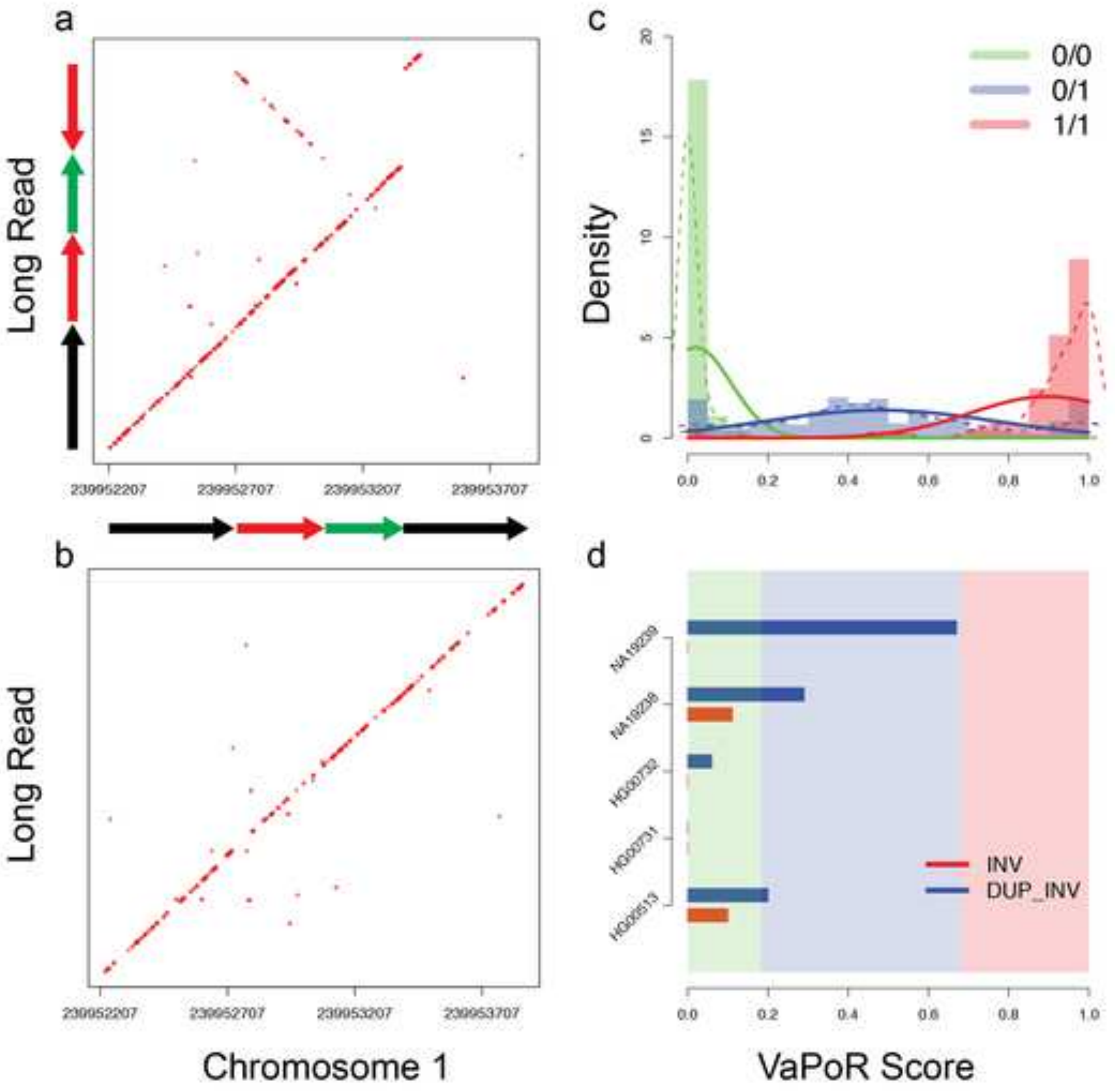

Supplementary Figures

Click here to access/download
Supplementary Material
supplementary_figures.docx

Supplementary Tables

Click here to access/download
Supplementary Material
supplementary_tables.xlsx
