# Supplementary material for: A recurrence-based approach for validating structural variation using long-read sequencing technology
Source: Gigascience. 2017 Jul 19;6(8):1–9. doi: 10.1093/gigascience/gix061 (PMC5737365; doi:10.1093/gigascience/gix061)

Supplementary Figure 1. Sensitivity and false discovery rate (FDR) of validating heterozygously simulated structural variants were calculated at different cutoffs set for VaPoR score. Sensitivity and FDR both decreases with the cutoff increasing, with >90% sensitivity and <10% FDR achieved at cutoff=0.1


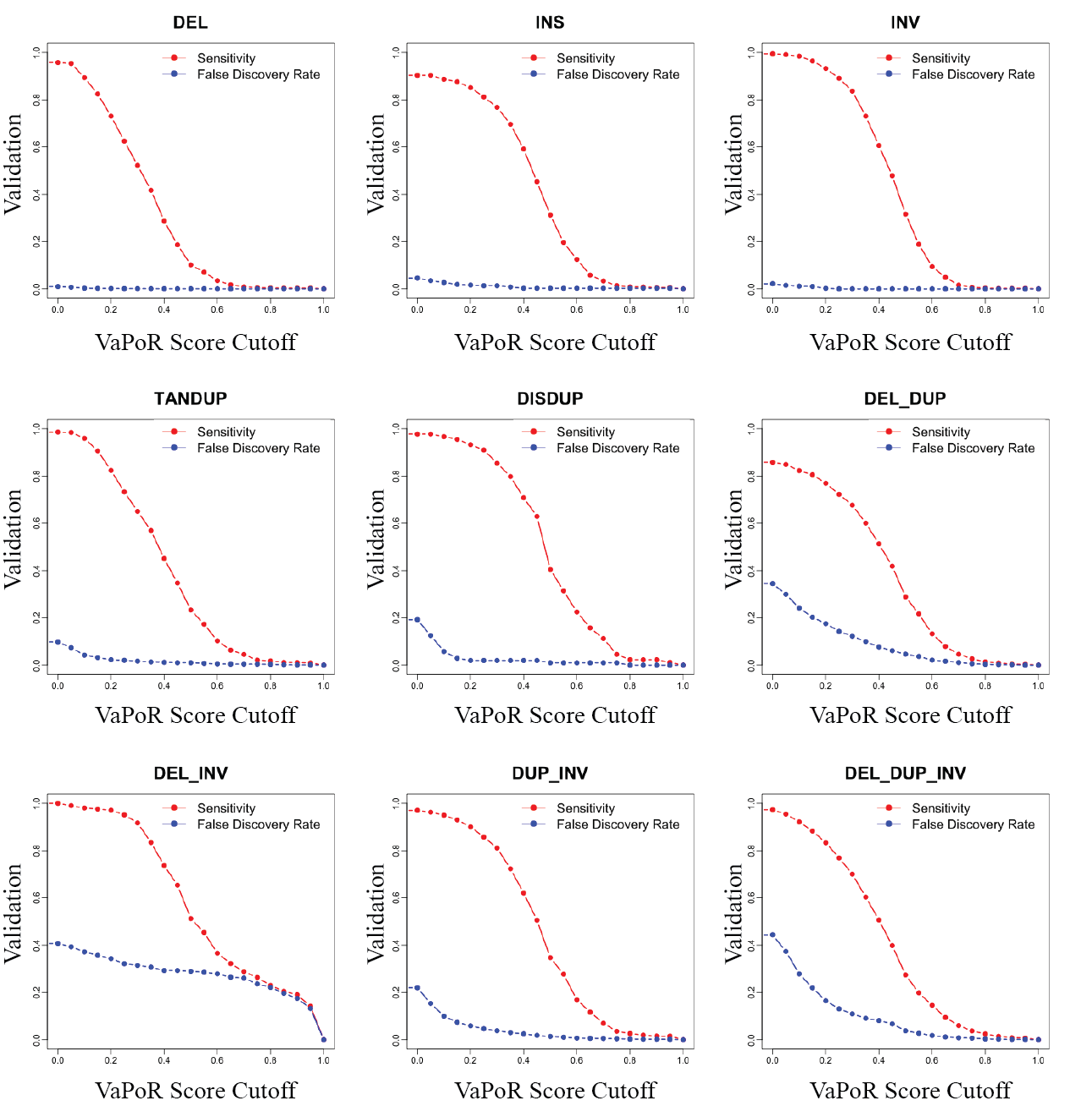


Supplementary Figure 2. Sensitivity and false discovery rate (FDR) of validating homozygously simulated structural variants were calculated at different cutoffs set for VaPoR score. Sensitivity and FDR both decreases with the cutoff increasing, with >90% sensitivity and <10% FDR achieved at cutoff=0.1 – 0.25


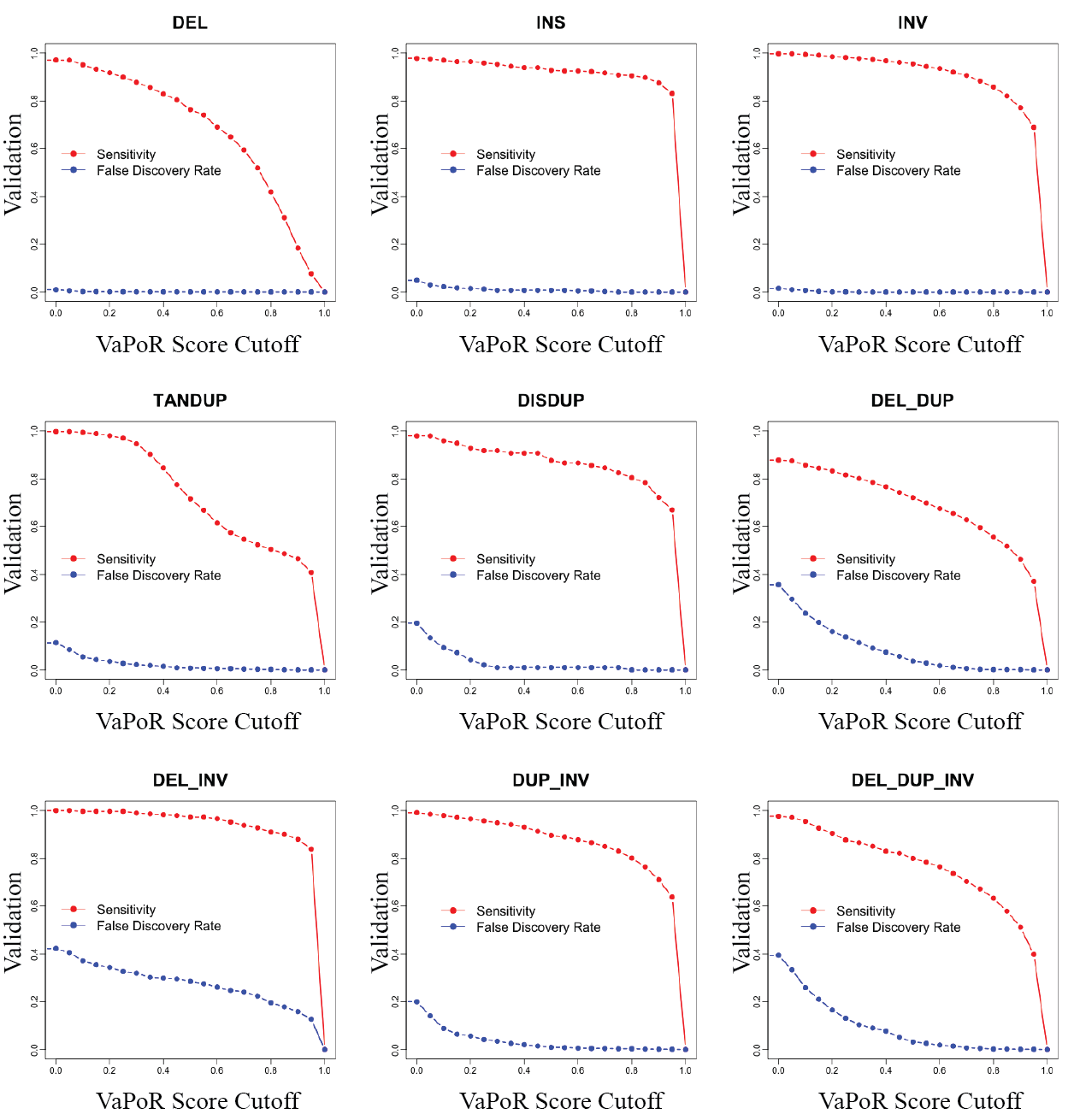


Supplementary Figure 3. Length distribution of PacBio reads in HG00513 (red) , and the corresponding distribution of SVs reported in the same sample (blue). The median length of aligned PacBio long reads is 15.6Kb, and ~5% of the 1KGP phase3 predictions in HG00513 have lengths over the median.

Supplementary Figure 4. An example of VaPoR on large SVs predictions that have few long sequences fully transverse. (a) is the IGV screenshot of the region on chr1: 72300660-72346132, which indicates a homozygous deletion of 45.5Kb. (b) is the recurrence plots of pacbio read (read name: m150923_001907_42216_c100828312550000001823180911251591_s1_p0/81227/27314_30304) versus reference sequence in the original and altered format

Supplementary Figure 5. Plot of validation rate when validating the simulated SVs with fake breakpoints deviated from the real ones by different bases. Validation rates are averaged from simulated deletion, insertion, inversion and tandem duplication at 30X coverage.


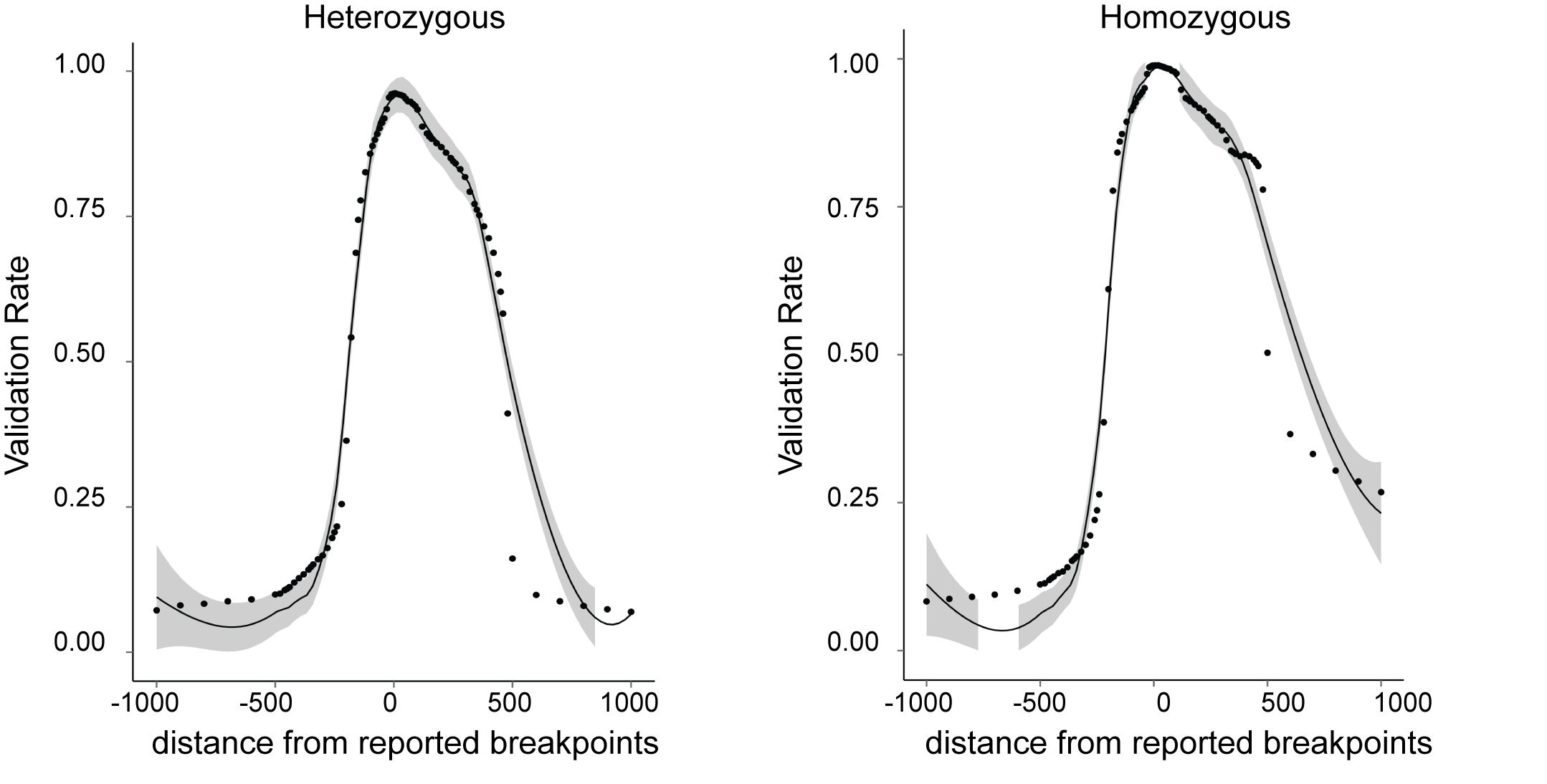


Supplementary Figure 6. Plot of validation rate when validating the simulated SVs with fake breakpoints deviated from the real ones by different bases. Validation rates are shown for simulated deletion, insertion, inversion and tandem duplication at 30X coverage.


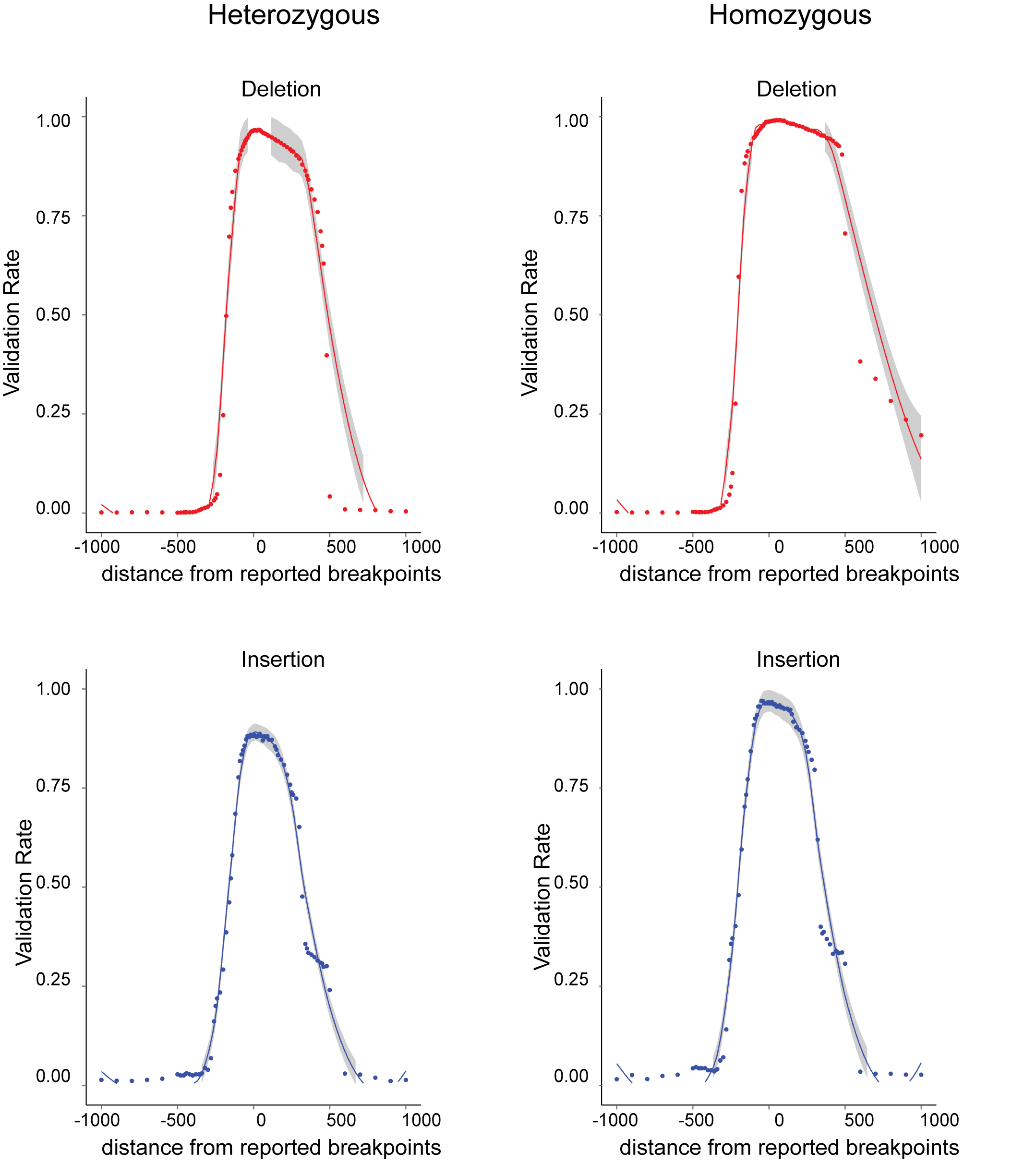


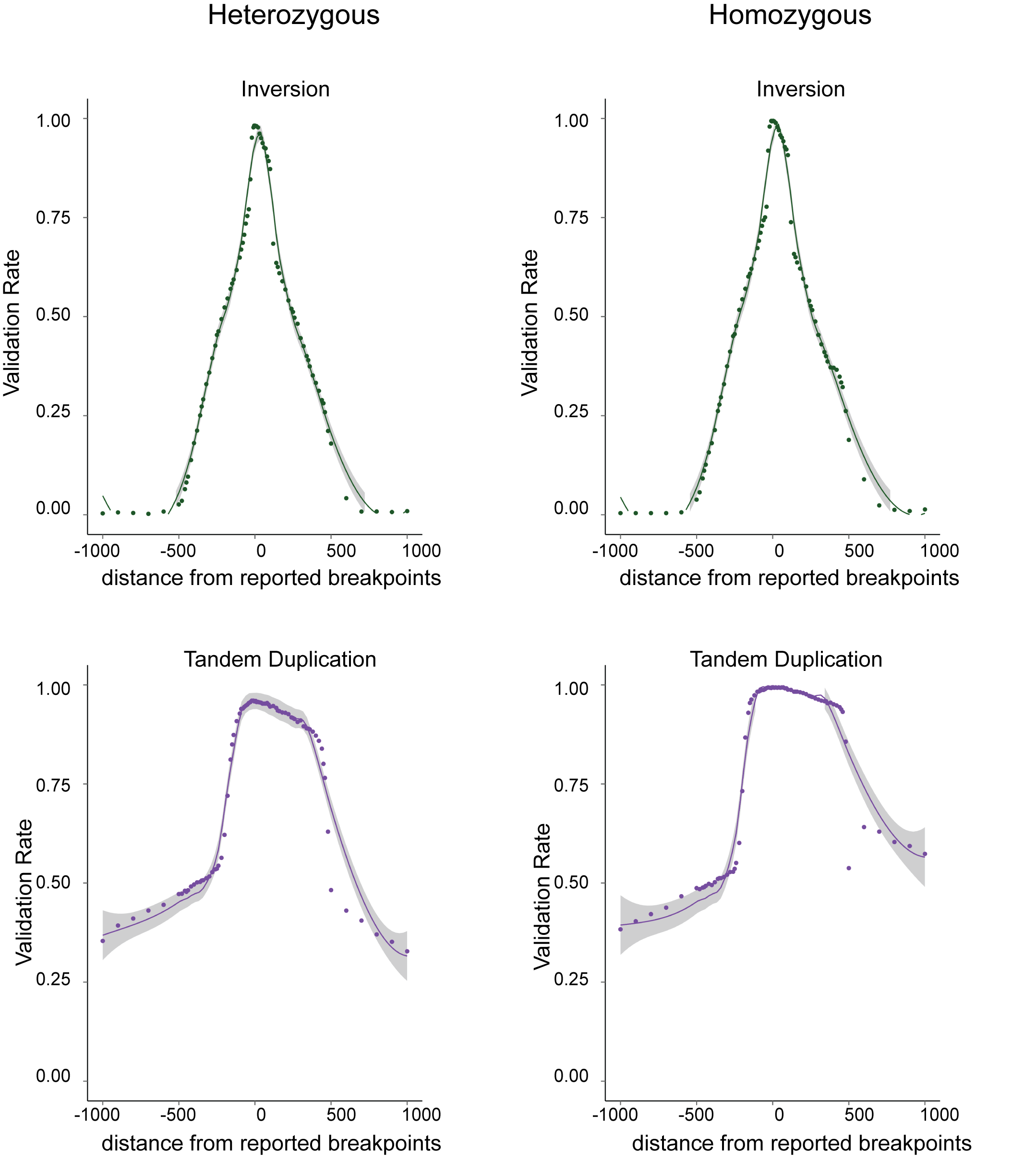


Supplementary Figure 7. Averaged run time (seconds) of each simulated SV summarized and plotted at different read depth. Simple and complex SVs are estimated separately, shown in red and blue lines respectively.


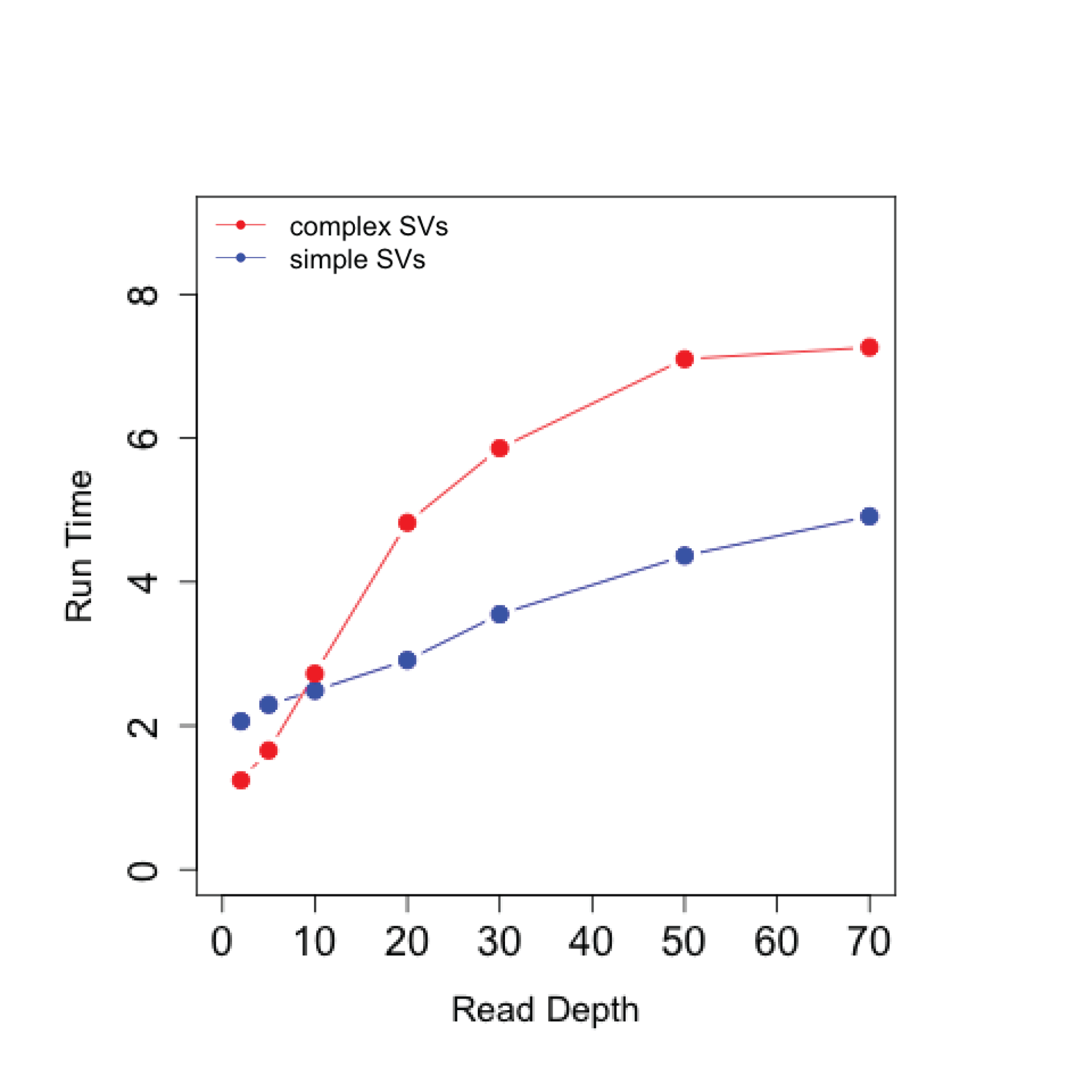

Supplement: Supplement Materials [file gix061_supp.zip › supplementary_figures.docx]
