# Supplementary material for: A recurrence-based approach for validating structural variation using long-read sequencing technology
Source: Gigascience. 2017 Jul 19;6(8):1–9. doi: 10.1093/gigascience/gix061 (PMC5737365; doi:10.1093/gigascience/gix061)
Supplement: Response-to-Reviewer-Comments_Original-Submission.pdf [file gix061_response-to-reviewer-comments_original-submission.pdf]

Author's Response To Reviewer Comments

Response to Reviewers

Reviewer #1: The method described here is of great value to the field. The lack of good truth sets plague SV detection, and the manual validation of thousands of SVs with PCR does not scale. Long reads can help here, and VaPoR puts forward a good framework for using this data.

***We thank Dr. Layer for his critical assessment of our manuscript, and agree that VaPoR will indeed provide some additional utility to ongoing and future SV studies.

Major issues:
In the context of disease analysis, correctly differentiating between a HET and a HOM ALT is nearly as important as validating the existence of the SV. The authors do address this to some extent by separating results for predicted HET and HOM ALT SVs, but it is not clear to me how to covert a VaPoR score to a genotype and if the true positive criteria required a matching genotyped. I would strongly suggest that the authors dig deeper into this issue and report the proportion of HETs that were correctly called HET, HETs called HOM ALT, etc. across read depths. This information would be of great value to readers that are considering long read validation.

***We agree, and have now implemented a framework (Li, 2011 pmid: 21903627) for deriving genotype likelihoods based on the number of supporting REF and ALT reads, and report a high concordance (non-HOMREF) to previous published results coupled with manual inspection (Supplemental Table 6). VaPoR also now reports both the ‘site’ validation score as well as the predicted genotype for each event.

It is not clear to me how VaPoR deals with imprecise breakpoints? Figure 3c,d give good results when the breakpoint is shifted +- 200bp. Does this mean that VaPoR can be used on breakpoints with at most a 200bp confidence interval? I think it is worth clearing this up considering that there are nearly 30K deletions (>50bp) in the 1000 genomes phase3 SV call set with non zero confidence intervals, and the mean size of those confidence intervals is >200bp. The commands I used to get to this result is below:

***The flexibility of VaPoR to validate offset breakpoints is somewhat dependent on the size of the flanking regions required, which has now been included as an additional parameter that can be adjusted based on the expected CI size of the SV prediction tool used to make the input predictions. Based on the phase3 call set, while the mean CI of breakpoints is indeed ~297bp, the median is only ~85bp suggesting that the majority of breakpoints are fairly accurate and the higher mean driven by a few outliers. Our high rate of validation overall on the phase3 set also suggests that these CIs may have been overestimated, in any case. We have included additional discussion of this in the manuscript under a new section entitled “SV Breakpoint Validation and Accuracy”.

Questions:
What are some examples of large variants with "few, if any, long reads that can traverse the

predicted SV"? How many variants are expected to be in this class?

***We compared the length distribution of the long reads with the SVs reported in the phase3 set and found that only ~5% of the SVs were longer than the median size of the long reads (~15Kbp). We have included this length comparison as a new Supplemental Figure 5. We also provide a case example in IGV of a deletion too large (>45Kbp) for an individual read to traverse and the corresponding dot plot from VaPoR as a new Supplemental Figure 6.

Can VaPoR be used to clean up the alignments of long reads around SVs? In my experience many alignments continue past the breakpoints leading to a large amount of noise. It would seem that this process could be used to correct some of this issues.

***This is an interesting question; in principle, the sections of the reads pertaining to other parts of the SV that cross the breakpoint could be assigned as a supplementary alignment based on their new relative positions from the VaPoR structure. For longer reads this may become more problematic as there may be multiple SVs covered. We feel this falls outside the current validation utility of VaPoR, however it is a good idea and we can consider including this as an additional feature in the future.

Reviewer #2: The authors describe a VaPoR, a tool for validating structural variants (SVs) using long reads. The described tool falls into a meta-category that does not directly predict structural variants and can not with absolute certainty validate them, rather which provides a prediction of the correctness of individual SVs. The authors use simulations, data from the thousand genomes project and data from a previous study (Layer et al.) to validate the correctness of their validations.

***We thank the reviewer for their critical assessment of our manuscript. We hope this version clarifies the methodology as well as better communicates the overall utility of the tool.

Comments:
(0) I could not really follow the description of the method. The authors use some unusual terminology to describe the method which they do not fully define. What is a "recurrence plot"? I think of a recurrence plot as described here: https://en.wikipedia.org/wiki/Recurrence_plot I don't think this is what the authors intended? The method writeup is also too imprecise. What does "A recurrence matrix is then derived by sliding a fixed-size window with 1bp step through each read to mark positions where the read and reference sequence are identical" mean? I think I know, but it would be much better if the authors more precisely defined their meaning. Similarly using very complex subscripted variables like "x_{i,k,s,R_x}" without defining them properly is egregious. Finally, the associated Figure 1 for the method is too complex - I could not follow what all the reads were doing or what the meaning of the different sequences of arrows marked "prediction" and "reference" mean?

***The use of the term ’recurrence’ refers to the spatial labeling of individual k-mers to their recurrent positions between two sequences, and our strategy is built upon the analysis of such recurrences. In terms of visualization, a ‘dot’ plot is actually a type of recurrence plot (see:

https://en.wikipedia.org/wiki/Dot_plot_(bioinformatics)), however we agree this may be overly obfuscated and so have changed ‘recurrence plot’ to ‘dot plot’ in the manuscript, while retaining ‘recurrence’ for the methodology.

***With regards to the methods description, we agree that what we presented was too imprecise and have modified our description to be more rigorous. We have also reworked and simplified Figure 1.

(1) Philosophically I struggle with the approach of VaPoR. As it can not provide a gold standard for validation, and does not output much evidence (it seems) with the associated VaPoR score it seems like it provides just another opinion, and one that can not be absolutely relied upon. I understand that it is useful to calculate a desirable but complex objective function on a prediction when that objective function can not easily be directly used in making the original prediction, often because the direct optimization is intractable, but I would be careful about selling such an objective function as a validation method. I would rather see the authors move in the direction of outputting their prediction and supporting evidence (e.g. supporting read alignments), in a manner that allows the VaPoR score to be interpreted as yet another source of evidence.

***We have modified the VaPoR output to include not only the VaPoR score but also the number of supporting read alignments for each allele (reference and prediction) to allow users to make decisions based on multiple points of evidence. We agree that VaPoR does not represent a conclusive validation of any single event by itself, but would note that the manual inspection of ‘dot’ plots is a long-standing approach for validation that has been recently used by the 1000 Genomes Project (Sudmant et al, 2015) and other projects using PacBio data (Chaisson et al, 2015; Huddleston et al, 2014, 2016). Although manual inspection may perform nominally better than our automated approach, we feel that the utility of scalability that VaPoR brings will be of use to the greater SV community.

(2) The validation seems okay. I am a bit skeptical about the run times of the tool, quoted at multiple seconds per variant. I think it would also be useful to state how long it takes to validate a complete genome.

***We agree, and now provide an estimate of whole genome analysis for a given coverage and typical SV set under “Runtime and efficiency”.

(3) The paper has many typos. It also has some very odd word choices that I do not think convey the authors meaning correctly.

***We have improved the overall wording and grammar in the manuscript and have hopefully fixed all of the typos.

(4) The code for the project should be linked prominently in the main manuscript.

***We have now included the URL to the GitHuB repository in the abstract.

Overall I think this is a valiant attempt to do something useful in the space of SV prediction, but

I think the paper needs to polish to improve communication.

***We again thank the reviewer for their assessment and hope we have alleviated their concerns with this newer, improved version.
