# Supplementary material for: A recurrence-based approach for validating structural variation using long-read sequencing technology
Source: Gigascience. 2017 Jul 19;6(8):1–9. doi: 10.1093/gigascience/gix061 (PMC5737365; doi:10.1093/gigascience/gix061)
Supplement: Response-to-Reviewer-Comments_Revision-1.pdf [file gix061_response-to-reviewer-comments_revision-1.pdf]

Author's Response To Reviewer Comments

Reviewer reports:

Reviewer #1: I think the paper is good to go. For what it is worth, I think Sup Figs 3 and 4 are the most informative and I would strongly suggest finding a way to move them to the main text.

***We thank the reviewer for their comments. We have now merged Supp Figs 3 and 4 and inserted them as a new Figure 2. We removed the 70X data point for aesthetics since it did not add any additional information that the 20X, 30X and 50X points did not already show.

Reviewer #2: Thanks for the revision. I am mostly happy with the revision, and appreciate that the authors have improved the readability of the manuscript. However, I remain concerned that the methods section is too obfuscated and complex. Firstly, while I accept the authors assertion that "recurrence matrix" is the general mathematical term, I'd appreciate the authors including "dot" plot in the methods section. I think the target audience here will appreciate that change. Secondly, and much more importantly, the section titled "Small Variants Assessment" still defines variables with subscripts that are not defined. What is i, k and s? Not trying to be difficult, but I want the method to be crystal clear!

***We thank the reviewer for their comments. We have added ‘in the form of a dot plot’ to the methods section to augment the recurrence analysis description. We also define the subscript variables in the text as well, specifically “For an SV k in sample s that is covered by n reads” (first sentence) and also define ‘i' as the index of summation from 1 to m and state “where m is the total number of records in the matrix” immediately after the first equation. We hope this clarifies the methodology.
