# Supplementary material for: A recurrence-based approach for validating structural variation using long-read sequencing technology
Source: Gigascience. 2017 Jul 19;6(8):1–9. doi: 10.1093/gigascience/gix061 (PMC5737365; doi:10.1093/gigascience/gix061)
Supplement: Reviewer-1-Report-(Original-Submission).pdf [file gix061_reviewer-1-report-(original-submission).pdf]

**Reviewer Report**

**Title:** A recurrence based approach for validating structural variation using long-read sequencing technology

**Version:** Original Submission **Date:** 4/18/2017

**Reviewer name:** Ryan Layer

**Reviewer Comments to Author:**

The method described here is of great value to the field. The lack of good truth sets plague SV detection, and the manual validation of thousands of SVs with PCR does not scale. Long reads can help here, and VaPoR puts forward a good framework for using this data.Major issues:In the context of disease analysis, correctly differentiating between a HET and a HOM ALT is nearly as important as validating the existence of the SV. The authors do address this to some extent by separating results for predicted HET and HOM ALT SVs, but it is not clear to me how to covert a VaPoR score to a genotype and if the true positive criteria required a matching genotyped. I would strongly suggest that the authors dig deeper into this issue and report the proportion of HETs that were correctly called HET, HETs called HOM ALT, etc. across read depths. This information would be of great value to readers that are considering long read validation.It is not clear to me how VaPoR deals with imprecise breakpoints? Figure 3c,d give good results when the breakpoint is shifted +- 200bp. Does this mean that VaPoR can be used on breakpoints with at most a 200bp confidence interval? I think it is worth clearing this up considering that there are nearly 30K deletions (>50bp) in the 1000 genomes phase3 SV call set with non zero confidence intervals, and the mean size of those confidence intervals is >200bp. The commands I used to get to this result is below:bcftools view -G ALL.wgs.integrated_sv_map_v2.20130502.svs.genotypes.vcf.gz \| bcftools query -f "%CHROM %POS %END %SVTYPE %CIEND %CIPOS\n" \| grep DEL \| awk '$3-$2>50' \| awk '$5 != "."' \| grep -v "0,0" \| tr ',' ' ' \| awk '{print -1*$5+$6,-1*$7+$8;}' \| tr ' ' '\n' \| meanQuestions:What are some examples of large variants with "few, if any, long reads that can traverse the predicted SV"? How many variants are expected to be in this class?Can VaPoR be used to clean up the alignments of long reads around SVs? In my experience many alignments continue past the breakpoints leading to a large amount of noise. It would seem that this process could be used to correct some of this issues.-Ryan Layer, University of Utah

**Level of Interest**

Please indicate how interesting you found the manuscript: An article of importance in its field

**Quality of Written English**

Please indicate the quality of language in the manuscript: Acceptable

**Declaration of Competing Interests**

Please complete a declaration of competing interests, considering the following questions:

- Have you in the past five years received reimbursements, fees, funding, or salary from an organisation that may in any way gain or lose financially from the publication of this manuscript, either now or in the future?
- Do you hold any stocks or shares in an organisation that may in any way gain or lose financially from the publication of this manuscript, either now or in the future?
- Do you hold or are you currently applying for any patents relating to the content of the manuscript?
- Have you received reimbursements, fees, funding, or salary from an organization that holds or has applied for patents relating to the content of the manuscript?
- Do you have any other financial competing interests?
- Do you have any non-financial competing interests in relation to this paper?

If you can answer no to all of the above, write 'I declare that I have no competing interests' below. If your reply is yes to any, please give details below.

I declare that I have no competing interests

I agree to the open peer review policy of the journal. I understand that my name will be included on my report to the authors and, if the manuscript is accepted for publication, my named report including any attachments I upload will be posted on the website along with the authors' responses. I agree for my report to be made available under an Open Access Creative Commons CC-BY license (http://creativecommons.org/licenses/by/4.0/). I understand that any comments which I do not wish to be included in my named report can be included as confidential comments to the editors, which will not be published.

I agree to the open peer review policy of the journal
