# Supplementary material for: A recurrence-based approach for validating structural variation using long-read sequencing technology
Source: Gigascience. 2017 Jul 19;6(8):1–9. doi: 10.1093/gigascience/gix061 (PMC5737365; doi:10.1093/gigascience/gix061)
Supplement: Reviewer-2-Report-(Original-Submission).pdf [file gix061_reviewer-2-report-(original-submission).pdf]

Reviewer Report

Title: A recurrence based approach for validating structural variation using long-read sequencing technology

Version: Original Submission **Date:** 4/23/2017

Reviewer name: Benedict Paten

Reviewer Comments to Author:

The authors describe a VaPoR, a tool for validating structural variants (SVs) using long reads.The described tool falls into a meta-category that does not directly predict structural variants and can not with absolute certainty validate them, rather which provides a prediction of the correctness of individual SVs.The authors use simulations, data from the thousand genomes project and data from a previous study (Layer et al.) to validate the correctness of their validations.Comments:(0) I could not really follow the description of the method. The authors use some unusual terminology to describe the method which they do not fully define. What is a "recurrence plot"? I think of a recurrence plot as described here: https://en.wikipedia.org/wiki/Recurrence_plot I don't think this is what the authors intended? The method writeup is also too imprecise. What does "A recurrence matrix is then derived by sliding a fixed-size window with 1bp step through each read to mark positions where the read and reference sequence are identical" mean? I think I know, but it would be much better if the authors more precisely defined their meaning. Similarly using very complex subscripted variables like "x_{i,k,s,R_x}" without defining them properly is egregious. Finally, the associated Figure 1 for the method is too complex - I could not follow what all the reads were doing or what the meaning of the different sequences of arrows marked "prediction" and "reference" mean?(1) Philosophically I struggle with the approach of VaPoR. As it can not provide a gold standard for validation, and does not output much evidence (it seems) with the associated VaPoR score it seems like it provides just another opinion, and one that can not be absolutely relied upon. I understand that it is useful to calculate a desirable but complex objective function on a prediction when that objective function can not easily be directly used in making the original prediction, often because the direct optimization is intractable, but I would be careful about selling such an objective function as a validation method. I would rather see the authors move in the direction of outputting their prediction and supporting evidence (e.g. supporting read alignments), in a manner that allows the VaPoR score to be interpreted as yet another source of evidence.(2) The validation seems okay. I am a bit skeptical about the run times of the tool, quoted at multiple seconds per variant. I think it would also be useful to state how long it takes to validate a complete genome.(3) The paper has many typos. It also has some very odd word choices that I do not think convey the authors meaning correctly.(4) The code for the project should be linked prominently in the main manuscript.Overall I think this is a valiant attempt to do something useful in the space of SV prediction, but I think the paper needs to polish to improve communication.

Level of Interest

Please indicate how interesting you found the manuscript: An article whose findings are important to those with closely related research interests

Quality of Written English

Please indicate the quality of language in the manuscript: Not suitable for publication unless extensively edited

Declaration of Competing Interests

Please complete a declaration of competing interests, considering the following questions:

- Have you in the past five years received reimbursements, fees, funding, or salary from an organisation that may in any way gain or lose financially from the publication of this manuscript, either now or in the future?
- Do you hold any stocks or shares in an organisation that may in any way gain or lose financially from the publication of this manuscript, either now or in the future?
- Do you hold or are you currently applying for any patents relating to the content of the manuscript?
- Have you received reimbursements, fees, funding, or salary from an organization that holds or has applied for patents relating to the content of the manuscript?
- Do you have any other financial competing interests?
- Do you have any non-financial competing interests in relation to this paper?

If you can answer no to all of the above, write 'I declare that I have no competing interests' below. If your reply is yes to any, please give details below.

None

I agree to the open peer review policy of the journal. I understand that my name will be included on my report to the authors and, if the manuscript is accepted for publication, my named report including any attachments I upload will be posted on the website along with the authors' responses. I agree for my report to be made available under an Open Access Creative Commons CC-BY license (http://creativecommons.org/licenses/by/4.0/). I understand that any comments which I do not wish to be included in my named report can be included as confidential comments to the editors, which will not be published.

I agree to the open peer review policy of the journal
