# Supplementary material for: A recurrence-based approach for validating structural variation using long-read sequencing technology
Source: Gigascience. 2017 Jul 19;6(8):1–9. doi: 10.1093/gigascience/gix061 (PMC5737365; doi:10.1093/gigascience/gix061)
Supplement: Reviewer-2-Report-(Revision-1).pdf [file gix061_reviewer-2-report-(revision-1).pdf]

**Reviewer Report**

**Title:** A recurrence based approach for validating structural variation using long-read sequencing technology

**Version:** Revision 1 **Date:** 6/18/2017

**Reviewer name:** Benedict Paten

**Reviewer Comments to Author:**

Thanks for the revision. I am mostly happy with the revision, and appreciate that the authors have improved the readability of the manuscript. However, I remain concern that the methods section is too obfuscated and complex. Firstly, while I accept the authors assertion that "recurrence matrix" is the general mathematical term, I'd appreciate the authors including "dot" plot in the methods section. I think the target audience here will appreciate that change. Secondly, and much more importantly, the section titled "Small Variants Assessment" still defines variables with subscripts that are not defined. What is i, k and s? Not trying to be difficult, but I want the method to be crystal clear!

**Level of Interest**

Please indicate how interesting you found the manuscript: An article whose findings are important to those with closely related research interests

**Quality of Written English**

Please indicate the quality of language in the manuscript: Needs some language corrections before being published

**Declaration of Competing Interests**

Please complete a declaration of competing interests, considering the following questions:

- Have you in the past five years received reimbursements, fees, funding, or salary from an organisation that may in any way gain or lose financially from the publication of this manuscript, either now or in the future?
- Do you hold any stocks or shares in an organisation that may in any way gain or lose financially from the publication of this manuscript, either now or in the future?
- Do you hold or are you currently applying for any patents relating to the content of the manuscript?
- Have you received reimbursements, fees, funding, or salary from an organization that holds or has applied for patents relating to the content of the manuscript?
- Do you have any other financial competing interests?
- Do you have any non-financial competing interests in relation to this paper?

If you can answer no to all of the above, write 'I declare that I have no competing interests' below. If your reply is yes to any, please give details below.

None

I agree to the open peer review policy of the journal. I understand that my name will be included on my report to the authors and, if the manuscript is accepted for publication, my named report including any attachments I upload will be posted on the website along with the authors' responses. I agree for my report to be made available under an Open Access Creative Commons CC-BY license (http://creativecommons.org/licenses/by/4.0/). I understand that any comments which I do not wish to be included in my named report can be included as confidential comments to the editors, which will not be published.

I agree to the open peer review policy of the journal
